# Parallels and discrepancies between non‐native species introductions and human migration

**DOI:** 10.1111/brv.70004

**Published:** 2025-02-20

**Authors:** Danish A. Ahmed, Ronaldo Sousa, Alejandro Bortolus, Ceray Aldemir, Nicole F. Angeli, Dagmara Błońska, Elizabeta Briski, J. Robert Britton, Carlos Cano‐Barbacil, Aaron Clark‐Ginsberg, Irina Culic, Ross N. Cuthbert, Jaimie Dick, Romina D. Dimarco, Franz Essl, Teun Everts, Emili García‐Berthou, Mathew Hauer, Antonín Kouba, Melina Kourantidou, Ulrich Kutschera, Stefano Mammola, Irene Martín‐Forés, Olivier Morissette, Martin A. Nuñez, Julian D. Olden, Lucian Pârvulescu, Jan Pergl, David Renault, Axel Eduardo Rico‐Sánchez, James C. Russell, Ismael Soto, Ali Serhan Tarkan, Tuğba Uçma Uysal, Hugo Verreycken, Lorenzo Vilizzi, Ryan Wasserman, Priscilla Wehi, Phillip J. Haubrock

**Affiliations:** ^1^ CAMB, Center for Applied Mathematics and Bioinformatics, Department of Mathematics and Natural Sciences Gulf University for Science and Technology Mubarak Al‐Abdullah Area/West Mishref Hawally 32093 Kuwait; ^2^ CBMA – Centre for Molecular and Environmental Biology/ARNET‐Aquatic Research Network/ IB‐S, Institute of Science and Innovation for Bio‐Sustainability, Department of Biology University of Minho Campus Gualtar Braga 4710‐057 Portugal; ^3^ Grupo de Ecología en Ambientes Costeros (GEAC), Instituto Patagónico para el Estudio de los Ecosistemas Continentales (IPEEC‐CONICET) Puerto Madryn Argentina; ^4^ Department of Public Administration, Faculty of Economics and Administrative Sciences Muğla Sıtkı Koçman University Muğla Türkiye; ^5^ Division of Fish and Wildlife Government of the Virgin Islands Frederiksted VI 0084 USA; ^6^ University of Lodz Faculty of Biology and Environmental Protection, Department of Ecology and Vertebrate Zoology Lodz 90‐237 Poland; ^7^ Department of Life and Environmental Sciences, Faculty of Science and Technology Bournemouth University Poole Dorset UK; ^8^ GEOMAR Helmholtz‐Zentrum für Ozeanforschung Kiel Kiel 24148 Germany; ^9^ Department of River Ecology and Conservation Senckenberg Research Institute and Natural History Frankfurt am Main Frankfurt 60325 Germany; ^10^ RAND Santa Monica CA USA; ^11^ Department of Sociology Babeș‐Bolyai University Cluj‐Napoca Romania; ^12^ Institute for Global Food Security, School of Biological Sciences, Queen's University Belfast Belfast UK; ^13^ Department of Biology and Biochemistry University of Houston Houston TX 77204 USA; ^14^ Grupo de Ecología de Poblaciones de Insectos, IFAB (INTA – CONICET) San Carlos de Bariloche Río Negro Argentina; ^15^ Division of BioInvasions, Global Change and Macroecology, Department of Botany and Biodiversity Research University of Vienna Rennweg 14 Vienna 1030 Austria; ^16^ Research Institute for Nature and Forest Genetic Diversity Geraardsbergen Belgium; ^17^ KU Leuven, Department of Biology Plant Conservation and Population Biology Heverlee Belgium; ^18^ GRECO, Institute of Aquatic Ecology, University of Girona Girona 17003 Spain; ^19^ Department of Sociology Center for Demography and Population Health, Florida State University 609 Bellamy Building, 113 Collegiate Loop Tallahassee Florida 32306‐2240 USA; ^20^ Faculty of Fisheries and Protection of Waters South Bohemian Research Center of Aquaculture and Biodiversity of Hydrocenoses, University of South Bohemia in České Budějovice Vodňany 389 25 Czech Republic; ^21^ Univ Brest, Ifremer, CNRS, IRD, UMR 6308, AMURE, IUEM Plouzane F‐29280 France; ^22^ Department of Sociology, Environmental and Business Economics University of Southern Denmark Degnevej 14 Esbjerg 6705 Denmark; ^23^ I‐Cultiver, Inc.,Manteca, CA 95336, USA & AK Evolutionsbiologie Freiburg i. Br 79104 Germany; ^24^ Molecular Ecology Group (MEG), Water Research Institute (IRSA), National Research Council (CNR) Largo Tonolli, 50 Pallanza 28922 Italy; ^25^ NBFC, National Biodiversity Future Center Palermo 90133 Italy; ^26^ Laboratory for Integrative Biodiversity Research (LIBRe) Finnish Museum of Natural History (LUOMUS), University of Helsinki Helsinki Finland; ^27^ School of Biological Sciences, The University of Adelaide Adelaide South Australia 5005 Australia; ^28^ Chaire de recherche sur les espèces aquatiques exploitées, Université du Québec à Chicoutimi Chicoutimi Quebec G7H 2B1 Canada; ^29^ School of Aquatic and Fishery Sciences, University of Washington Seattle WA 98195 USA; ^30^ Crayfish Research Centre, Institute for Advanced Environmental Research, West University of Timisoara Oituz 4 Timisoara 300086 Romania; ^31^ Department of Biology, Faculty of Chemistry, Biology, Geography West University of Timisoara Pestalozzi 16A Timisoara 300115 Romania; ^32^ Institute of Botany CAS Průhonice Czech Republic; ^33^ UMR CNRS 6553 ECOBIO [Ecosystèmes, biodiversité, évolution], Université Rennes avenue Général Leclerc Rennes cedex 35042 France; ^34^ Posgrado en Hidrociencias, Colegio de Postgraduados Texcoco Estado de México Mexico; ^35^ School of Biological Sciences, University of Auckland New Zealand; ^36^ Department of Basic Sciences, Faculty of Fisheries Muğla Sıtkı Koçman University Muğla Türkiye; ^37^ Department of International Trade and Finance, Faculty of Economics and Administrative Sciences Muğla Sıtkı Koçman University Muğla Türkiye; ^38^ Research Institute for Nature and Forest, Monitoring and Restoration of Aquatic Fauna Linkebeek Belgium; ^39^ Department of Biological Sciences College of Science, Research Center for the Natural and Applied Sciences, The Graduate School, University of Santo Tomas Manila Metro Manila 1008 Philippines; ^40^ Department of Zoology and Entomology Rhodes University Makhanda South Africa; ^41^ South African Institute for Aquatic Biodiversity Makhanda South Africa; ^42^ Centre for Sustainability, University of Otago Dunedin New Zealand; ^43^ CAMB, Center for Applied Mathematics and Bioinformatics, Gulf University for Science and Technology Mubarak Al‐Abdullah Kuwait

**Keywords:** biosecurity, cultural assimilation, ethnocentrism, ecological resilience, globalisation, sociopolitical dynamics, transdisciplinary research

## Abstract

Biological invasions and human migrations have increased globally due to socio‐economic drivers and environmental factors that have enhanced cultural, economic, and geographic connectivity. Both processes involve the movement, establishment, and spread of species, yet unfold within fundamentally different philosophical, social and biological contexts. Hence, studying biological invasions (invasion science) and human migration (migration studies) presents complex parallels that are potentially fruitful to explore. Here, we examined nuanced parallels and differences between these two phenomena, integrating historical, socio‐political, and ethical perspectives. Our review underscores the need for context‐specific approaches in policymaking and governance to address effectively the challenges and opportunities of human migration and harm from biological invasions. We suggest that approaches to studying the drivers of biological invasions and human migration provide an excellent opportunity for transdisciplinary research; one that acknowledges the complexities and potential insights from both fields of study. Ultimately, integrating natural and social sciences offers a promising avenue for enriching the understanding of invasion biology and migration dynamics while pursuing just, equitable, and sustainable solutions. However, while human migration is a clear driver of biological invasions, drawing on principles from biological invasions to understand past and current human migration risks oversimplification and the potential for harmful generalisations that disregard the intrinsic rights and cultural dynamics of human migrations. By doing so, we provide insights and frameworks to support the development of context‐specific policies that respect human dignity, foster cultural diversity, and address migration challenges in ways that promote global cooperation and justice. This interdisciplinary approach highlights the potential for transdisciplinary research that acknowledges complexities in both fields, ultimately enriching our understanding of invasion biology and migration dynamics while pursuing equitable and sustainable solutions.

## I. INTRODUCTION

Many core principles and fundamental theories in invasion science have been intensively explored in recent decades (Jeschke & Heger, 2018; Daly *et al*., 2023) and are generally not considered transferable to the complexities surrounding human migrations. Repeatedly throughout human history, however, politicians have misused scientific terminology and principles to advance ideologically based policies. For instance, Adolf Hitler purposely misapplied Charles Darwin's theories, later referred to as ‘Social Darwinism’, to support his racist ideologies (Gould, 1996; Weikart, 2013). More recently, scientists explicitly advocate against the use of xenophobic concepts when describing biological invasions – the movement of (non‐native) species by any means, generally mediated by or resulting from human activities beyond their native range (Simberloff, 2003). However, the rhetoric used by invasion scientists, particularly the use of terms like ‘invasion’ or ‘invasive’, which evoke ideas of foreignness and threats, has been misused by activists, journalists, media pundits, social influencers, and politicians from all sides of the political spectrum (Schlaepfer, Sax & Olden, 2011; Sax, Schlaepfer & Olden, 2022). These groups have inappropriately paralleled the concept of biological invasions with the fear of immigrants, as seen in political discourse in the USA, where such rhetoric has fuelled xenophobic sentiment (Subramaniam, 2001; Pitoski, Lampoltshammer & Parycek, 2021). This includes terms like ‘alien’ (exotic, non‐native, non‐indigenous) and ‘invasive’ (i.e. non‐native species that spread beyond the introduction point), which are widely used by invasion scientists (Soto *et al*., 2024a).

This overlap between metaphorical language and invasion science terminology, often criticised for its militaristic origin (Dawkins & Krebs, 1979; Guareschi *et al*., 2024), has been argued to promote human prejudices and xenophobia (Simberloff, 2003; Tassin & Kull, 2015; Fall, 2017). This can influence not just the terminology used, but also the cultural perspectives shaping discourses. Part of the historical background on the rhetoric of ‘invasions’ concerning human immigration in the USA has deep roots, tracing back to at least the 19th century, decades before the term ‘invasive species’ was coined to refer to a subset of problematic non‐native species. This theme began to surface prominently with an 1873 advertisement in the *San Francisco Chronicle*, which announced the ‘Chinese Invasion’, claiming that 900,000 immigrants were arriving to America from China. This hyperbolic warning was a prelude to the Chinese Exclusion Act of 1882, America's first law banning a specific national group, influenced by fears stirred by such ‘invasion’ rhetoric. Over time, this metaphor of invasion expanded to include not just Chinese but also other Asian immigrants from Japan and Korea, then Southern and Eastern Europeans, and by the 20th century encompassing immigrants from Mexico and other Latin American countries. This invasion rhetoric has been a fixture in American political discourse (and in many other countries), often used to provoke fear or justify restrictive immigration policies (Zimmer, 2019). In South America, Argentine governments in the late 1800s made great efforts to attract millions of rural workers by advertising in European newspapers and lobbying in different countries. Although their rhetoric preferred Europeans over the ‘gauchos' (people of European descent born in the Americas), ‘creoles' (skilled horsemen from Argentina, Uruguay, and Brazil), and Indigenous people, the majority of the European migrants suffered open discrimination and abuse from a nationalist society that tended to see this massive immigration as an ‘invasion’ threatening their local values and way of life (see Gargurevich, 1994). Through a historical and ecological lens, the parallels between invasion science and historical immigration policies demonstrate the existence of a shared metaphorical framework, albeit one with potential for misleading analogies (Pincetl, 2007), which may be inappropriate and cause confusion.

Metaphorical language is shaping perceptions towards immigration, reinforcing divisive and exclusionary ideologies. Similarly, the politicisation of scientific language illustrates how terms originally intended for solely scientific contexts are repurposed in political rhetoric, shaping public and policy discussions about immigration (Vogelaar, 2021). The strategic misuse of, e.g. militaristic, language aims to be emotive and to influence public perception and justify policy decisions, highlighting the need for careful consideration of language in both public discourse and science. Dangerous rhetoric continues to manifest in modern times. For instance, migrants and asylum seekers are often portrayed as invaders attempting to breach Europe's borders, undermining its cultural, social, political, and economic stability (Toivanen, 2019). More recently, President Donald J. Trump (Republican) in the USA referred to migrants as harmful when saying that Democrats wanted migrants to ‘infest our country’, and Marine Le Pen (National Rally) in France described immigration as a ‘migrant invasion’. In April 2024, the leaders of the Vox party urged the Spanish government to ‘block the illegal immigration invasion that threatens Spain’. Similarly, the Australian Kevin Rudd (Labor) referred to people‐smuggling into the country as a ‘scourge’ and Bob Carr (Labor) declared that Sydney was ‘full’ back in the year 2000. Tony Blair's (Labour) policies in Great Britain, as well as many other European politicians from a variety of different political affiliations (see the recent examples of hate speech and discrimination toward subcultures in Italy; Vannacci, 2023), occasionally emphasised ‘control measures’ around illegal immigration (Mulvey, 2010).

Despite superficial similarities, applying invasion science principles, terminologies, and theories to human migration risks oversimplifying both their complexities (Scholten, 2022) and potentially dehumanising migrants by neglecting their sentience, intrinsic rights, individuality, values, and societal contributions, thereby reflecting a (largely negative) value‐laden understanding of human movement (Coates, 2007). This comparison is often leveraged by those constructing narratives against human migration; yet, scholars can recognise the fundamental differences between biological invasions and human migration, questioning the scientific basis for such analogies. While similarities in the ethics of both human migration and species translocations were recently highlighted (Switzer & Angeli, 2016), distinctions based on ethical, biological, and social differences have not been thoroughly explored in the interdisciplinary literature. In this context, we examine here the popular use of invasion science terminology, by e.g. journalists, activists, and politicians, rather than its technical and scientific application, by e.g. scientists. Considering the need for a more integrative framework that better articulates perspectives from the social sciences (McNeely, 2001; Bortolus & Schwindt, 2022), this study therefore aims to (*i*) identify and discuss nuanced parallels and highlight crucial differences between the study of non‐native species introductions and human migration, and (*ii*) underscore the potential negative repercussions of hasty and superficial generalisations and misused terminology.

## II. COMPARING NON‐NATIVE SPECIES INTRODUCTIONS AND HUMAN MIGRATION

### (1) Non‐native species introductions

Since the age of exploration (Crosby, 2004), during the colonial era (Coates, 2007), or when planned as part of statebuilding (Maxwell‐Stewart, Inwood & Stankovich, 2015; Godfrey, 2019), the movement of domesticated or wild organisms with people across continents was unregulated. It was often incentivised and driven by the colonists' desire to impose their vision of ‘improvement’ on both the environment and the colonised peoples, exploit resources and dominate newly colonised lands by establishing familiar species from the old homeland (driven by acclimatisation societies), and to bring ‘strange’ peculiarities home (Lenzner *et al*., 2022; Muñoz‐Mas *et al*., 2023). This led to widespread introductions of non‐native species, including crops and livestock, which profoundly altered native ecosystems and agricultural practices in both the homeland and the colonised regions (Cuthbert *et al*., 2023; Soto *et al*., 2024b; Turbelin *et al*., 2024). Today, globalisation has become a major driver of non‐native species introductions (Seebens *et al*., 2017) and the introduction of non‐native organisms from any biological kingdom is recognised to pose major threats to biodiversity, ecosystems, and human societies (Reynolds & Aldridge, 2021). This causes substantial economic damage through, e.g. agricultural losses, infrastructure degradation, and, among others, control costs (Diagne *et al*., 2021; Gallardo *et al*., 2024). The study of non‐native species dates back to the late 18th and early 19th centuries when the effects of species introductions were first described by Pehr Kalm, a Swedish botanist and disciple of Carl N. Linnaeus (Gottschalk, 1966), the French naturalist Alcide d'Orbigny, and Charles Darwin (Ludsin & Wolfe, 2001; Kutschera & Khanna, 2024). The formal field of invasion science was, however, only established in the late 20th century, following the works of Charles S. Elton and the publication of his book *The Ecology of Invasions by Animals and Plants* (Elton, 1958). Later, the issue of biological invasions was addressed by the Scientific Committee on Problems of the Environment (SCOPE) programme, an international group of 22 science unions and 40 national committees that collaborate on issues pertaining to the environment (White, 1987).

Human activities create pathways for non‐native species introductions. If environmental conditions and propagule pressure (i.e. introduction effort, as a composite measure of the number of attempts made and the amount of individuals released into a non‐native region; Lockwood, Cassey & Blackburn, 2005) are sufficient, then populations of non‐native species may establish and proliferate (Jeschke & Strayer, 2005; Milanović *et al*., 2020). Once these populations grow in number, they eventually spread from the initial location of introduction (Richardson *et al*., 2000) and may cause measurable socio‐economic or ecological impacts including, among others, monetary damage and the loss of biodiversity (Simberloff *et al*., 2012), hence being deemed as ‘invasive’ (Soto *et al*., 2024b).

**Fig. 1:**
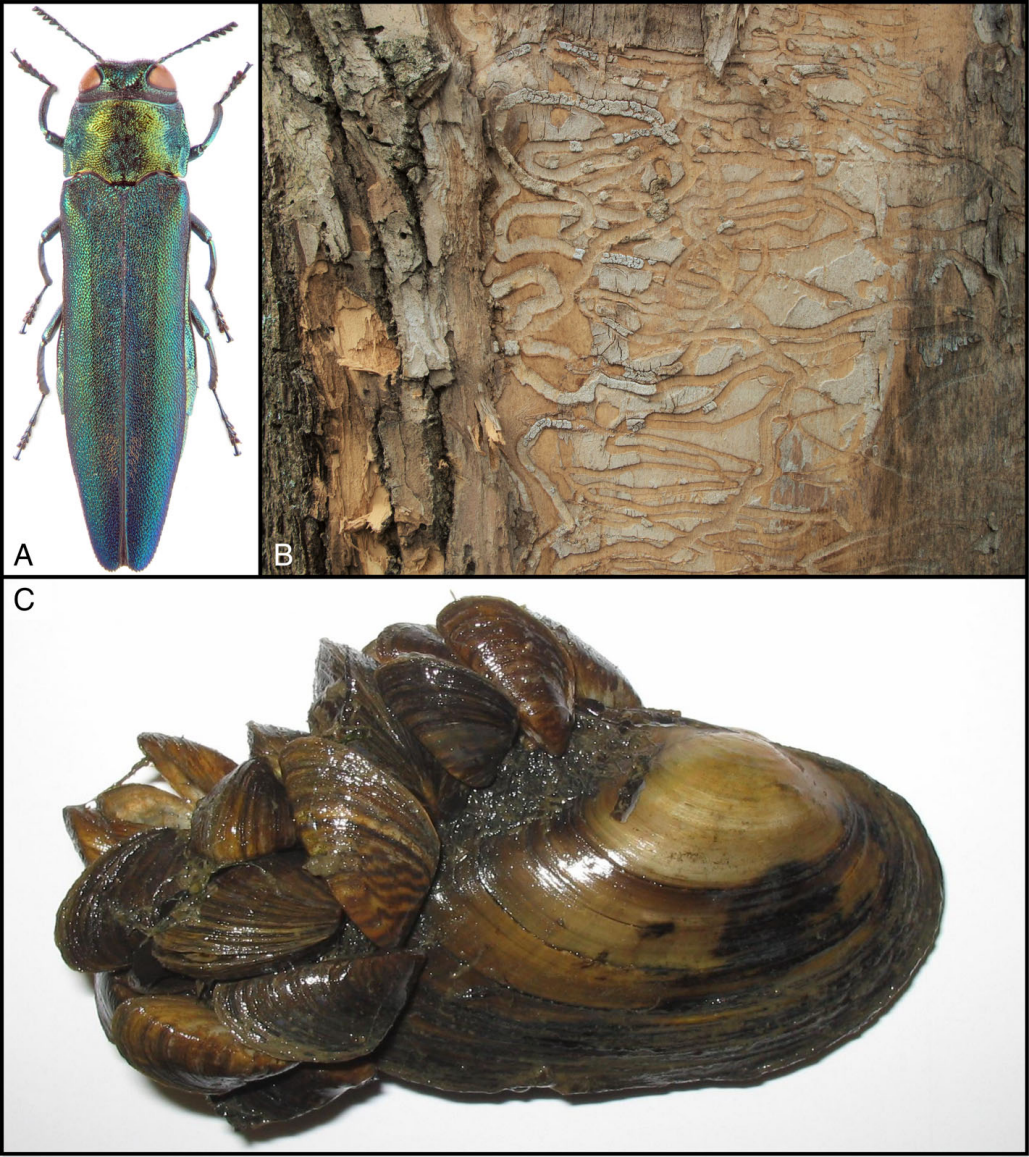
(A) Emerald ash borer *Agrilus planipennis* (specimen collected on European ash *Fraxinus excelsior* in Moscow Region, Russia, on 15 July, 2006; size 10.5 mm (photograph by K. V. Makarov, with permission). (B) Galleries of *A. planipennis* larvae under bark of a dead ash tree *F. excelsior* in Moscow region, Russia, 2016 (photograph by D. L. Musolin). (C) The duck mussel *Anodonta anatina* heavily infested by the zebra mussel *Dreissena polymorpha* (photograph by Ronaldo Sousa).

Over time, non‐native species introductions may result in a permanent alteration (or deterioration) of the invaded native communities (Del Rio‐Hortega *et al*., 2022) and ecosystems (Bortolus, Carlton & Schwindt, 2015; Emery‐Butcher, Beatty & Robson, 2020). Non‐native species may also affect human health and well‐being, detrimentally impacting socio‐cultural dimensions including, but not limited to, monetary losses (Ehrenfeld, 2010; Diagne *et al*., 2021). The introduction of non‐native species is studied by invasion scientists to understand their spread dynamics and impacts, but also to identify novel ways to predict, prevent (by controlling the introduction and spread), contain, suppress, and/or eradicate non‐native populations, thereby mitigating their impacts (Britton *et al*., 2023). For example, the emerald ash borer *Agrilus planipennis* (Fig. 1A,B) is a beetle species that has turned from an inconspicuous East Asian species into one of the most devastating pests of ash trees in the world. It has destroyed millions of trees in North America, European Russia, and Ukraine, and currently threatens central and western European ash trees in forests and urban areas (Kirichenko *et al*., 2021; Musolin *et al*., 2022; Sun *et al*., 2024). Another example is the zebra mussel *Dreissena polymorpha*, which was introduced from the Ponto‐Caspian region to North America through ships' ballast water, prompting global regulations on ballast water transport and decontamination (Ricciardi & MacIsaac, 2000). This species can severely impact native bivalve populations (Sousa, Pilotto & Aldridge, 2011), many of which are threatened (Lopes‐Lima *et al*., 2018). *Dreissena polymorpha* competes with native mussels for food and space and can attach to their shells (Fig. 1C), impeding their feeding and movement, including burrowing in the soft substrate during times of water scarcity and droughts to which native species are more sensitive (Sousa *et al*., 2011). Additionally, the cane toad *Rhinella marina* and buffel grass *Cenchrus ciliaris* are both among the most problematic introduced species in Australia; however, they were originally brought to the country to control crop‐infesting beetles (Shine, 2010) and for pasture production, respectively (Marshall, Lewis & Ostendorf, 2012).

**Fig. 2:**
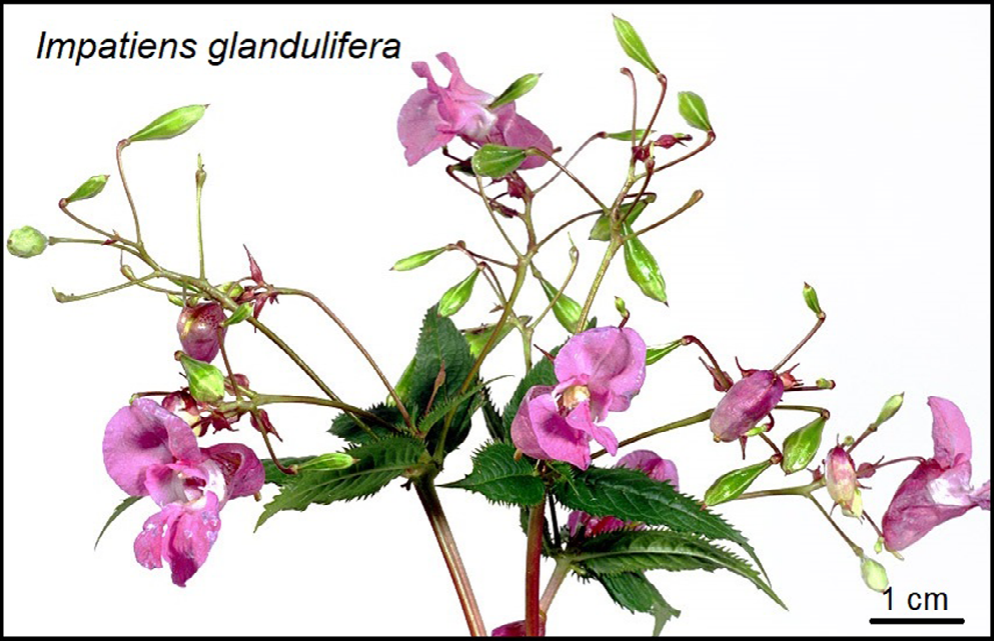
Flowering individual of the Himalayan balsam *Impatiens glandulifera*, an ornamental plant introduced to Europe from Asia in 1840, where it escaped from botanical gardens into surrounding areas, and has since established dense populations. This non‐native species can dominate moist habitats such as tall herb vegetation along rivers, where it may outcompete native plants. Original image by U. Kutschera.

Among invasion scientists, there is a broad consensus that the negative effects of non‐native species introductions generally outweigh the benefits they present (Carneiro *et al*., 2024), while acknowledging the importance of several non‐native species for economic enterprises and human well‐being (Sax *et al*., 2022). Indeed, in particular cases, non‐native species can provide some benefits (Sax *et al*., 2022) or even coexist due to adaptation and co‐evolution between the established non‐native species and the recipient ecosystem (Schlaepfer *et al*., 2010; Martín‐Forés *et al*., 2016; Martín‐Forés, Guerin & Lowe, 2017). The negative impacts of non‐native species can vary over time, space, and stakeholder groups, and depending on the context and species involved (Simberloff *et al*., 2024). Successfully proliferating non‐native species can, due to rapid growth and reproduction, be seen as a readily available source of food (e.g. protein; Haubrock *et al*., 2021) or other goods (e.g. wood combustion), especially in economically disadvantaged regions (Iyer *et al*., 2021). In the past, they have been argued to enrich native biodiversity by, e.g. acclimatisation societies (Lockwood, Hoopes & Marchetti, 2013). Some non‐native species have also become valuable to agriculture, forestry, hunting, fishing, or are seen to benefit aesthetics, being an integral part of economies and food provisioning (e.g. mammals introduced as livestock; Clout & Russell, 2008). Proponents of non‐native species sometimes argue that these species can enhance species richness and ecosystem diversity (Thomas, 2013; Schlaepfer, 2018), often overlooking indirect consequences such as alterations in vegetation community structure (Guerin *et al*., 2019) or increased plasticity for introduced populations in the invaded ranges compared to native ones (Martín‐Forés *et al*., 2018a,b). Alternatively, advocates may not recognise the potential negative impacts on native ecosystems if non‐native species escape beyond confinement (Simberloff *et al*., 2012). Another argument of proponents of non‐native species might be their prolonged presence at low abundances or biomass, i.e. in a stage not posing any threat to native species and ecosystems. However, it has been shown that shifting environmental conditions can trigger rapid population growth, transforming a non‐native species from harmless or benign to highly impactful (Spear *et al*., 2021). A prominent example of a non‐native species with diverging values among stakeholders is the spread of the Himalayan balsam *Impatiens glandulifera* in Europe (Fig. 2). The introduction of this large ornamental plant is associated with both negative effects on soil, fungal compositions, and native plants, as well as potential benefits (e.g. facilitating the establishment and spread of pollinators).

### (2) Human migration

There is a vast difference in how scholars from various social science disciplines – such as economics, political science, international relations, security studies, anthropology, sociology, post‐colonial studies, and feminist studies – approach the study of migration. These differences arise because each discipline begins with distinct ontological and epistemological premises. While there have been attempts to integrate these diverse perspectives, they have been met with limited success (Portes, 1997; Faist, 1998; Brettell & Hollifield, 2000; Glick Schiller, 2008). One element that distinguishes among theories of migration is the unit of analysis, particularly concerning the agency of the immigrant and how the decision to migrate is made. The neoclassical theory of migration (Kurekova, 2011) takes the rational individual as its unit of analysis, asserting that the decision to migrate is made by weighing pros and cons (push–pull factors) according to classic methodological individualism. Human capital theory (Fleischhauer, 2007) extends this approach by examining the convertibility rates of individuals' abilities and capacities in the new society, thereby assessing potential success. This approach, viewing human migration as a fundamental aspect of human nature (Rizvi, 2022), emphasises individual agency and the pursuit of comfort and economic well‐being, and has profoundly shaped societies and cultures throughout history (Fisher, 2013; Niedenthal *et al*., 2019). The new economics of migration, on the other hand, takes the household as its unit of analysis. It posits that the decision to migrate is made collectively by the household, generally to reduce risks and diversify opportunities for the entire family (Stark & Bloom, 1985; Abreu, 2012). This approach implies various strategies where some individuals may migrate while others remain at home, supporting each other mutually. This perspective influences the understanding of integration and the calculation of a migration's impact. Immanuel Wallerstein's world‐systems theory introduces structural factors as the main determinants of immigration, using the nation‐state as the unit of analysis. In this theory, the movement of capital across borders in a highly globalised economy characterised by unequal exchanges forces labour from peripheral and semi‐peripheral regions to migrate to core states (Massey *et al*., 1993).

In the literature, the concepts of ‘immigration’ and ‘migration’ are often used interchangeably, despite their distinct meanings and implications. Immigration is the process of moving (e.g. to a foreign country), with a focus on the destination aspect of migration (Brettell & Hollifield, 2022). Migration often indicates the relocation of individuals from one location to another, frequently – but not necessarily – crossing political borders, with the intention of establishing permanent or long‐term residency (Castles, de Haas & Miller, 2014; Wallerstein, 2015). Migration is the definition and unit of analysis utilised in this review. Following Fairchild's (1925) typology, human migration across national borders for the purpose of settling in a different country (Kaitmazova & Caberti, 2016) is classified into (*i*) economic migration (including both high‐ and low‐skilled migrants moving to find economic prosperity), (*ii*) family reunification (migrants moving to join family members who have already settled abroad), (*iii*) forced migration (including refugees and asylum seekers managed under the 1951 Refugee Convention), and (*iv*) student migration (encompassing significant numbers of students moving in pursuit of higher education). Moreover, migration can be classified by duration. Temporary migration encompasses workers (and international interns) migrating for seasonal or short‐term work assignments that contribute to the migration of ideas or, in certain cases, ‘semi‐forced labour’, while permanent migration characterises the long‐term or indefinite relocation of individuals or groups to a new country or region, often involving formal processes such as obtaining residency or citizenship. Migration can, however, also be classified as irregular if it occurs outside legal frameworks and regulations as in the case of overstaying visas and human smuggling (Bales, 2012; Freeman, 2017).

Human migration is generally driven by human needs and the will to thrive (often referred to as ‘human nature’; Rizvi, 2022) by seeking economic opportunities or fleeing political instability and conflicts. Many of these drivers are directly influenced by human actions and policies (Castelli, 2018; Czaika & Reinprecht, 2022), but generally involve the movement of people across natural or political borders. Large‐scale human migrations, often referred to as ‘mass migrations’ (i.e. excluding, e.g. the migration of individuals for academic, expat, student, and other comparable purposes), despite not being clearly defined by migration scholars (Abramitzky, Boustan & Eriksson, 2014), started around 2 million years ago with *Homo erectus* spreading across continents, followed by various archaic humans and eventually modern *Homo sapiens* around 70,000–50,000 years ago (Posth *et al*., 2016; Saltré *et al*., 2024). These modern humans expanded into Eurasia, interbred with local populations such as Neanderthals and Denisovans, and eventually reached remote areas like the Americas 20,000 years ago and Oceania within the last 2,000 years (Spriggs, 2011; Villanea & Schraiber, 2018). Migrations also continuously occurred throughout ancient times, including the Indo‐European Migrations (Shevoroshkin, 1986; Andersen, 2003), the Greek Colonisation (Donnellan, 2016) and, among others, the Migration Period during the later stages of the Roman Empire (Goffart, 2006; De Ligt & Tacoma, 2016). The transatlantic slave trade stands as the most extensive coerced human migration across oceans in recorded history (Schrover, 2022). These migrations have profoundly shaped the fate of our planet, impacting not only human societies but also natural ecosystems and biodiversity (Sponsel, 2013). They have led to the emergence of diverse cultures, languages, and traditions, contributing to the rich tapestry of human civilisation we know today (McAuliffe & Khadria, 2020). As such, historical migrations have to be distinguished from, for example, migrations between the First and Second World War, post‐World War II migratory movements of, for instance, Europeans to Australia and South America, and ultimately major migration flows that appeared after 2010 following the events of the Arab Spring (Ongley & Pearson, 1995; Bani Salameh, 2019).

Throughout history, individuals have been compelled to relocate due to disasters (triggered by both natural and human‐derived hazards), the depletion of resources, or military defeats (Eltis & Richardson, 2008). During the transatlantic slave trade, an estimated 10–12 million Africans were forcibly transported as slaves to the Americas by European traders (Palmer, 1994; Walvin, 2013), with African rulers and merchants from many nations participating in capturing and selling such human commodity (Bennett, 2018; Fage, 2019). Approximately 5 million Africans were sent to Brazil, 2–3 million to the Caribbean, and roughly 450,000 to North America, where slave owners, despite constituting only a small minority of the people, included primarily white Europeans, but also black individuals and Native Americans (Rodrigues, 1962; Marques, 2019). Besides these tremendous numbers, many more died before being sold into slavery and during the transatlantic transport (Palmer, 1994). On the other hand, the economic role of these slaves evolved significantly as the commercial landscape of the New World transformed. Migrant slaves working outside the household became much more numerous and economically significant as the commercial transformation proceeded. Plantation, agriculture, and mining in North America, staffed predominantly by slaves, emerged as dominant economic activities in the 17th and 18th centuries. Unlike the rhetoric of ‘invasion’ used to stir fear and justify restrictive immigration policies in the 19th and 20th centuries, the transatlantic slave trade of earlier centuries was rationalised under the guise of economic necessity and racial superiority, highlighting a starkly different and morally abhorrent justification (Wilson, 1996; Williams, 2013). While having shaped the cultural nature of, for example, the USA (Davis, 1988), Brazil and Argentina (Borucki, Eltis & Wheat, 2015), this has also led to a common association of slavery with these specific forms of labour organisation.

In fact, slavery was historically considered a normal institution across various cultures worldwide (Newman, 2022). The moral and ethical revolution that recognised slavery as inherently wrong emerged prominently in the West, particularly with the abolitionist movements led by republicans in the USA, culminating in the abolition of slavery in the 19th century (Miers, 2011). Even at the peak of the West African slave trade, the exact number of Africans transported across the Atlantic to work in various sectors remains uncertain. What is known is the connection between the group of people belonging to the larger ethno‐linguistic family known as the ‘Slavs’ in Eastern Europe and the word ‘slave’, which emerged in the early mediaeval period when many Slavs were captured and enslaved by various European powers, as well as Muslim traders. This led to the adoption of the term *sclavus* in Medieval Latin to mean a person in servitude (Kłosowska, 2020; Štefan, 2021) as about 8 million people were moved between the 17th and 19th centuries (McNeill, 1984). The social and economic inequalities perpetuated by historic slave trades and colonialism have had long‐lasting impacts on human migration patterns, a phenomenon later described as ‘super‐wicked’ (Levin *et al*., 2012; Pitoski *et al*., 2021). Nowadays, slavery, albeit illegal throughout the world, still persists in a more elusive way, with more than 27 million people still trapped in one of history's oldest social conundrums (Bales, 2012) and 50 million people subjected to modern slavery according to the global slavery index (https://www.walkfree.org/global-slavery-index; Walk Free, 2023), a vast percentage of whom are migrants (Baker, 2019).

The extraction of resources and exploitation of labour in colonised countries created significant socio‐economic disparities, driving mass migrations both during and after the colonial period (Heywood, 2017). Post‐colonial states often struggle with economic instability and political turmoil, exacerbated by the legacies of colonial exploitation and uneven development, further fuelling migration (Gutiérrez Rodríguez, 2018). Moreover, continued exploitation by wealthy nations through unfair trade practices, debt imposition, and resource extraction perpetuates these inequalities, leading to ongoing human displacement and migration (Gutiérrez Rodríguez, 2018). Consequently, humans in these regions may want to migrate to more economically favourable regions or countries. However, their ability to migrate is often limited by inadequate access to humanitarian aid (van Houte, Kaşlı & Leerkes, 2023) or subjected to exploitation by human traffickers (e.g. in the Mediterranean; Achilli, 2016). Upon arriving at the intended destination (which may not always be the next safe country; Popescu, 2016), migrants may receive varying levels of assistance from local communities. These communities are typically expected to assist with their settlement by providing resources and access to the social system (Gutiérrez Rodríguez, 2018). The recipient society's willingness to help can be motivated by various factors, including relational ‘warm glow’, historic guilt, a high degree of altruism, the need to fill workforce and labour market shortages or to gain political leverage (Stoll, 2009). For instance, a study by Dustmann & Frattini (2014) on the economic consequences of immigration in the UK revealed that immigration policies have been significantly shaped by governmental needs and the specifically the need to address labour shortages in critical sectors such as healthcare and construction. Their analysis showed that these economic motivations can sometimes overshadow altruistic or historical reasons for accepting migrants, emphasising a strategic approach to immigration that may benefit the host country's economy (Dustmann & Frattini, 2014).

Overall, any given aid, which arguably differs among societies, comes at the price of the expectation of the migrant's will to integrate (by adapting to the new cultural environment). Over multiple generations, integration leads to assimilation in terms of values, behaviours, cultural norms, and, in some cases, religious beliefs – whether fully or partially (*sensu* Hirsch, 1942) – into the recipient society (Gordon, 2015). This anticipated integration does not always succeed and success rates may differ substantially among ethnic groups (see e.g. Kogan, Fong & Reitz, 2020), recipient societies, and the historical setting (Benitez, 2004; Anghel, 2012), ultimately being modulated by the number of migrating people, their culture and composition (see e.g. Statham & Tillie, 2016; Salikutluk & Menke, 2021), and the recipient society's socio‐cultural conditions (Anghel, 2012).

### (3) Similarities

At first glance, the principles underlying the introduction of non‐native species and human migration can appear similar due to the movement of organisms or individuals and the pivotal role of human actions as enablers or drivers (Table 1). Both processes may also involve the establishment of newcomers, whether they are non‐native species or foreign people, who may spread and, depending on their origin, culture, or biological parameters and the surrounding context, integrate into the new environment to varying degrees. In both cases, future climatic and land use changes are predicted to play a major role, either by enhancing the likelihood that non‐native species will establish and spread elsewhere (e.g. Townhill *et al*., 2017) or by increasing the hardship and decreasing the quality of life for humans in some regions (by e.g. decreasing access to fresh water; Mukheibir, 2010) and increasing incentives to migrate (Hermans & McLeman, 2021; Smirnov *et al*., 2023).

**Table 1:** Relevant terminology and their similarities and dissimilarities in the context of biological invasions and human migration.

Phenomenon	Conceptualisation	Similarities	Dissimilarities
Movement	The relocation of organisms (non‐native species) or individuals (human migrants) to new areas.	Both involve movement across borders, often driven by human activities and eventually resulting in the establishment of newcomers.	Non‐native species are often relocated unintentionally or intentionally for economic or aesthetic purposes, whereas human migration is driven by individual or collective intentional or unintentional decisions for economic, political, religious and/or safety reasons.
Establishment	The process by which newcomers integrate into the new environment and procreate.	Success depends on the match between the newcomer's traits and the new environment.	Non‐native species establishment can be purely ecological (i.e. governed by biotic and abiotic factors), whereas human migrants face social, cultural, political, and economic integration challenges.
Impact	The effects on the receiving environment or society.	Both can have significant impacts, positive or negative.	The most notable impacts of non‐native species are often ecological and socio‐economic, affecting local ecosystems and economies. By contrast, the impacts of human migration are primarily felt in social, cultural, economic, and political dimensions, influencing communities, cultures, and governance structures.
Ethics	The moral implications of the introduction or migration.	Both involve ethical considerations regarding the control, regulation or management of their movement.	Non‐native species management prioritises ecological balance and biodiversity, whereas human migration involves complex human rights issues, the sentience of individuals, and questions of fairness and justice within a community.
Terminology	The language used to describe the phenomenon, including expressions and concepts.	Both use value‐laden terms like ‘invasion’, ‘establishment’, and ‘control’, which can shape public perception.	While these terms have specific scientific meaning in ecology, misuse of scientific terms like ‘invasion’ in human migration contexts can dehumanise migrants.
Historical context	The historical background and development of the phenomenon.	Historical and technological events have shaped the movement of both non‐native species and human migrants.	Species introductions typically occurred within an ecological or colonial context, whereas the historical context of human migration often involved forced mass movements (e.g. slavery or fleeing war) in partial or complete absence of justice and with significant socio‐political consequences.
Integration	Observed patterns (in time and space) and theoretical frameworks explaining how newcomers integrate.	Ecological integration in species introductions can be compared to how human migration fills vacant societal roles, suggesting parallels in how both ecosystems and societies adapt to new arrivals.	Species integration focuses on ecological niches and interactions, whereas human integration models involve social, economic, and cultural dimensions.
Management and policy	Strategies and regulations to handle the phenomenon.	Both require management to mitigate negative impacts and enhance positive outcomes.	Non‐native species management focuses on prevention, control, containment, and eradication, whereas policies for human migration often prioritise legal status, social integration, and humanitarian aid.

Anthropological and sociological frameworks often describe human migration through push–pull dynamics, where adverse conditions in the origin region (e.g. conflict, economic hardship, or environmental degradation) ‘push’ individuals to leave, while attractive conditions at the destination (e.g. economic opportunities, safety, or better living conditions) ‘pull’ them in. These dynamics closely mirror ecological processes governing species invasions because, next to human activities, environmental pressures like habitat degradation or climate change ‘push’ species out of their native range, while better resource availability, lower competition, or predator release in new ecosystems ‘pull’ them towards successful establishment. Moreover, while both processes may involve discussions around ethical considerations, such as open borders and equal moral treatment, these principles do not apply uniformly. In the context of human migration, some advocate for the right to free movement and emphasise the unjust nature of exclusion based solely on origin, promoting assessments of impacts based on actual effects rather than origin (Davis *et al*., 2011). However, regarding non‐native species, regulations and ethical considerations often diverge significantly. Many regions and countries have strict legal frameworks, such as the European Union Regulation 1143/2014, designed to prevent the introduction, establishment, and spread of specific non‐native species deemed potentially harmful. In these cases, equal moral treatment and the right to move freely are not extended to all organisms, as conservation and ecological integrity are prioritised over unrestricted movement. Integration can be seen as a process that requires time and becomes frustrating or even harmful for both sides if not successful, with long‐lasting legacy effects that shape the ecological, social, or societal balance of the new ecosystem or society (Benitez, 2004). This is because, arguably, any newly arriving entity is not directly an integrated part of the new environment, but in some cases may have rapid detectable effects in the case of introduced non‐native species (Simberloff *et al*., 2012) or cause religious and cultural shifts as well as socio‐economic changes over time in the case of humans. Just as in the study of non‐native species introductions, the number of translocating individuals – whether propagule pressure for species or the scale of human migration – plays a crucial role in determining whether the presence of newcomers potentially modulates the degree of change, positive or negative, in the recipient environment (Koopmans, 2010). This outcome can be further influenced by cultural differences between the human migrant and the recipient society as stark cultural differences (or if migrants reject the cultures of host societies) increase the likelihood of conflicts (Watters, 2011). However, underlining the importance of ‘intent’, human migrants are sometimes intentionally attracted to fill key labour gaps, similar to non‐native species that have been seen as a way to fulfil lost ecosystem functions due to environmental changes and excessive human alterations to ecosystems (Svenning *et al*., 2016).

Human well‐being and similarly the integration of migrants and their impact on native societies can be based on four main categories (MEA, 2005): (*i*) safety; (*ii*) material and immaterial assets; (*iii*) health; and (*iv*) social, spiritual and cultural relations. The impact of non‐native species on these socio‐economic aspects is part of the proposed Socio‐economic Impact Classification of Alien Taxa (SEICAT; Bacher *et al*., 2018) and potentially also SEICAT+ (Vimercati *et al*., 2020). The principle of this system is to work with the freedom of choice and action, i.e. the opportunity to be able to achieve what a person values doing and being. However, EICAT (Blackburn *et al*., 2014) and EICAT+ (Vimercati *et al*., 2022) are already standard ways of classifying the environmental impact of non‐native taxa and can hardly be transferred to the context of migrants. Conversely, the SEICAT and SEICAT+, which are based on impact on human society, could be used for both non‐native taxa and migrants.

#### (a) Common origins

The apparent similarities between human migration and the introduction of non‐native species can be traced back to relevant, yet outdated economic theories like the Concentric Ring Theory of urban development (Brown, 2002). This theory, formulated by Ernest Burgess as part of the Chicago School of Sociology in the early 20th century, suggested that urban areas develop in a series of concentric rings that extend outward from the city centre. According to this theory, the central business district is at the core, surrounded by transitional zones that house poorer, immigrant, or ethnic groups. As these groups move in, they push the original, often wealthier inhabitants outward to more suburban rings. This process, described as ‘invasion’ and ‘succession’, portrays the central areas as continually being overtaken by newer, economically disadvantaged residents who are characterised as less ‘hardy’ due to their socio‐economic status (Splansky, 1966; Park & Burgess, 2019). Indeed, this model resembles the introduction, spread, and impacts of non‐native species in ecological systems, as both processes involve newcomers establishing themselves and potentially displacing the original occupants, leading to a reconfiguration of the existing system. However, while the displacement of native species by introduced ones is regarded as forced and negative, the movement of pre‐existing human populations due to immigration is voluntary and planned, and often includes material gains from the rents of their former properties [e.g. the emergence of *conventillos* or tenement houses in Argentina; Gargurevich, 1994; Benitez, 2004].

Throughout the past, there have been numerous attempts to draw analogies between human migration and non‐native species invasions (Simberloff, 2003). This has perpetuated the use of ‘invasion’ terminology to describe human movement and fostered a perception of similarities driven by inherent human tendencies to recognise patterns, even though such a linkage is tenuous at best. While humans are biologically a single cosmopolitan species, cultural identities, which transcend political borders, challenge generalisations based solely on geographic origin. This can be exemplified by the subdivision of Germany into East and West Germany and even lower, state‐level partitioning (van Hoorn & Maseland, 2010), but also by political, ethnic and ecological boundaries existing on the African continent (Dallimer & Strange, 2015). These unique cultural identities highlight the diversity within the human species and hamper most generalisations at the species level. Comparable recent works suggest that the intrinsic operative importance in the study of non‐native species introductions is at the population level (Haubrock *et al*., 2024). This means that while humans as a species have become cosmopolitan, they should not be dehumanised by being described as ‘invasive’ organisms in any part of the world (Utych, 2018). Rather, it should be acknowledged that humans have allegedly originated in Africa (Hershkovitz *et al*., 2018; but see also Stringer, 2020; Wolpoff, 2020; Bergström *et al*., 2021). Early hominins later spread and interbred with other hominin species such as Neanderthals in Europe and Denisovans in Asia (Stringer, 2020), technically becoming – after millions of years – an established non‐native species in most regions, including the African continent (Eswaran, Harpending & Rogers, 2005; Marean, 2015), and having arguably inflicted substantial damage to the environment (exemplified in the extinction of megafauna during the Pleistocene; Barnosky *et al*., 2004). The spread of humanity has nevertheless resulted in the emergence of diverse cultural identities globally, which must be considered and respected in discussions about migration and its effects on recipient societies.

#### (b) Integration into new environments

Another example of a shallow similarity is the process of integration. When human groups of different cultural backgrounds coexist, a process called ‘acculturation’ exists. Acculturation is the process of mutual influence and adaptation among cultural groups. Typically, this process is imbalanced, with the receiving group usually being numerically dominant and playing the prevailing role. According to the model of acculturation proposed by Berry (2017), individuals may adopt one of four strategies: integration, assimilation, separation, or marginalisation. This depends not only on their own views on the value of preserving their cultural identity and maintaining relationships with the larger society, but also on the attitudes, policies, and behaviours of the receiving society. The recipient society's openness to diversity, inclusiveness, and support for multiculturalism, or conversely, its discriminatory practices and exclusionary policies, can significantly influence which acculturation strategy is most viable or desirable for newcomers. Thus, acculturation is a dynamic and reciprocal process, shaped by both the individual's choices and the broader social context within the host society. These can be compared to terminology used for non‐native species (Tables 2 and 3; Blackburn *et al*., 2011), as well as to the hypotheses around ‘biotic resistance’, whereby the characteristics of the invaded environment alongside non‐native species traits influence establishment success. Piontkowski's Concordance Model (Piontkowski, Rohmann & Florack, 2002) further explores how acculturation attitudes can lead to different social outcomes, such as consensual (harmonious coexistence), conflictual, culturally problematic, and contact problematic states. According to this positivist rational‐choice theoretical model, the intent of the migrating group is crucial: if both groups favour integration, it leads to a consensual and harmonious state. Conversely, if the dominant group prefers assimilation while the migrating group seeks to preserve their identity (separation), it can lead to high conflict and problematic social interactions. This model also posits that if the dominant group prefers exclusion while the non‐dominant group seeks integration, or if both groups prefer segregation, different conflictual states can arise. High conflict may occur if the non‐dominant group prefers marginalisation while the dominant group aims for any form of contact, underscoring the importance of aligning acculturation attitudes to foster successful integration and minimise social tensions. Notably, unlike humans, non‐human species do not base their decisions or ecological interactions (e.g. competition, predation, mutualism) on what we refer to as ‘rational choices’ (Elster, 1986). However, the integration of intentionally introduced non‐native species into ecological communities may not go as planned and can result in ecological surprises, such as in the case of classical biological control introductions using generalist predators (e.g. cane toads; Shine, 2010; Shine, Ward‐Fear & Brown, 2020).

**Table 2:** Comparable terminology between non‐native species introductions (after Blackburn *et al.*, 2011) and international human migrations, highlighting why any comparison is to be considered as controversial and problematic.

Non‐native species introductions		Category	International human migrations	
Definition	Term		Term	Definition
Movement of species into a new region	Transport	Stage	International migration	Movement of people across national borders, temporarily or permanently, voluntarily or involuntarily, for a variety of reasons
Release of species into a new location	Introduction		Immigration	Arrival of people in a state with the intention to remain for a period of time (usually more than 1 year)
Species' survival and reproduction in a new location	Establishment		Integration	Mutual accommodation of immigrants and residents involving economic mobility and social inclusion
Expansion of species' range in the new region	Spread		Spatial dispersion	Settlement and internal migration patterns of immigrants into a state and their next generations migrating to new regions
Species becoming part of the local ecosystem	Establishment and interacting with native species	Outcome	Economic and social integration	Immigrants enter the labour market with varying outcomes – some achieve economic parity, while others face barriers like discrimination or credential recognition issues. Economic integration can result in both assimilation and economic marginalisation
Invasive species becoming self‐sustaining in the new location	Naturalisation		Naturalisation/assimilation	Process of voluntarily applying and becoming a citizen of the immigration country/immigrants become similar to the majority population in norms, values, behaviours, and characteristics over time
Species unable to survive or reproduce	Failed invasions		Multiple migration/return migration	Processes involving moves to more than one destination country. May include temporary migration, onward migration, return migration, and re‐migration. Return migration is situated anywhere on the voluntary‐forced axis, including repatriation, assisted return, removal, deportation
Rapid population growth followed by sharp decline	Boom–bust			
Measures to stop species from being introduced to new locations	Prevention	Management	Border control and security	Logistic and infrastructural measures to prevent entry, including fences, surveillance, intelligence and biometric data collection, pre‐authorisation and point of entry management
Restricting species to prevent further spread	Containment		Immigration policy	Immigration laws, rules, governmental decisions and directives regulating conditions of access, the right to remain, pathways to citizenship, and the application and adjudication of asylum. They also establish implementation procedures, quotas and caps for immigration
Reducing negative impacts of invasive species	Mitigation		Integration and social services	Language and education support programs, employment assistance, etc.
Complete removal of invasive species from a location	Eradication		Deportation/population transfer or resettlement	Removal as a result of an expulsion order/mass migration imposed by a state or an international authority

**Table 3:** Comparing the framework to categorise the pathways of non‐native species introductions from Hulme *et al.* (2008) to types of international human migration.

Non‐native species introductions			Human migration		
Initial introduction into region	Pathway	Definition	Definition	Type	Process
Commodity	Release	Intentional introduction as a commodity for release	Selection of immigrants for settlement according to a set of criteria	Selective immigration	Immigration programs
	Escape	Intentional introduction as a commodity but escapes unintentionally	Admission of foreigners for exercising economic activities in the receiving country. The period of time and type of employment are usually restricted. If their dependents are admitted, they too belong to this category. Receiving states may provide pathways for permanent settlement	Labour migration	Bilateral state agreements/ immigration legislation
	Contaminant	Unintentional introduction with a specific commodity	Admission of foreigners for education purposes. They may also provide qualified work and may be recruited for permanent settlement	International academic mobility	Selection by academic institutions/ Immigration legislation
Vector	Stowaway	Unintentional introduction attached to or within a transport vector	Temporary or permanent relocation of retired persons, typically to destinations with favourable climates	Retirement migration	Immigration legislation provisions
Dispersal	Corridor	Unintentional introduction *via* human infrastructures linking previously unconnected regions	Movement of persons across borders that happens following economic incentives, or possibly outside the regulatory frameworks of the sending, transit or receiving countries	Irregular migration	Immigration regulations of sending and receiving states
	Unaided	Unintentional introduction through natural dispersal of non‐native species across political borders	Involuntary, coerced movement of groups of people as a result of armed conflict, endemic violence, or climate change. People at high risk or victims of systematic or generalised violations of their human rights	Forced migration (including refugees; displaced persons; victims of traffic or slave trade migration)	Involuntary migration. Particular legislation is applied to process their removal, relocation or settlement

The principles of island biogeography (i.e. importance of area and isolation explaining species diversity in terms of immigration and extinction on islands) and range expansion can offer valuable insights into patterns of arrival, establishment, and spread of both biological invasions (Mollison, 1986; Moser *et al*., 2018) and human migration in new environments (Whittaker & Fernández‐Palacios, 2007; Whittaker, Fernández‐Palacios & Matthews, 2023). This is because key factors like dispersal ability and carrying capacity can shape the outcomes of both ecological and societal integration. For instance, the relative isolation and available area of a recipient environment – whether an actual island ecosystem or a socially or culturally insular community – can influence the likelihood of new arrivals successfully establishing themselves. Moreover, dispersal ability determines the capacity to overcome environmental barriers, which is similarly critical for both biological invasions and human migration. In ecological systems, successful non‐native species often demonstrate high dispersal rates or adaptations that facilitate spread, while in human migration, technological advancements and social networks can serve analogous functions, aiding individuals in navigating geographic and socio‐political barriers. Carrying capacity also provides a shared conceptual framework, as it represents the environmental or societal threshold for sustaining new arrivals without negative consequences.

Migration is often followed by a form of integration, although the processes may be complex and may also bring changes to the host society. However, not all migration results in integration as some instances may lead to segregation or conflict instead. The risks accompanying unsuccessful integration often lie in the socio‐economic, cultural, and linguistic challenges host societies face in integrating immigrants, particularly when there is a strong emphasis on preserving their own heritage and identity without adequately considering those brought by immigrants (Oliver & Gidley, 2015). It can also be seen in the case of newcomers, as humans seek comfort in familiar cultural and social practices (including within new surroundings), often forming tight‐knit communities that can help them navigate the challenges of adapting to a new environment while preserving their heritage and identity (Portes & Zhou, 1993; Sun, Chen & Xie, 2022). One may draw very simple parallels between invasion science and human migration. For example, one can argue that similar to non‐native species that require an environmental match to establish successfully (Martín‐Forés *et al*., 2015; Casado *et al*., 2015), immigration and integration are easier processes when cultural and climatic conditions are comparable to the country of origin (Soto Nishimura & Czaika, 2024). For instance, during the Great Migration of Europeans to Argentina in the late 1800s, Spanish immigrants found it easier to integrate than Italians and Eastern Europeans, as Argentinians were more familiar with the Spanish way of life and language (Benitez, 2004).

The effects of immigration on the receiving society are complex and may be perceived as positive or negative across different dimensions, such as health outcomes [e.g. spread of diseases or access to healthcare (Diallo, 2004; O'Donnell *et al*., 2016)], economic impacts [e.g. job creation or unemployment (Nathan, 2014; Taylor *et al*., 2016)], cultural influences (e.g. cultural exchange or erosion of traditions), and social relations [e.g. integration or social tension (Justino *et al*., 1998; Van Oudenhoven, Ward & Masgoret, 2006)]. The latter has recently garnered substantial attention, as poor integration may facilitate successive immigrants to move into the same regions that can, under certain instances, result in the creation of ethnic or cultural enclaves and subcultures that exacerbate existing problems (Milani, 2020; Kogan *et al*., 2020; Achard, 2022). Moreover, once established in the host society, a significant presence of migrants can lead to cultural and economic changes if integration is not successful. These changes may include negative reactions from the host society, shifts in labour markets, and impacts on social services and evolving community dynamics, akin to the complex impacts seen following the introduction of non‐native species (Russell & Kaiser‐Bunbury, 2019). According to the dual labour market theory, there is a marked division between jobs that offer good returns to education, are well paid, offer advancement opportunities and involve significant investment, and jobs that require low skills, are poorly paid, easily dispensable, and offer few career prospects. Mobility between the two is highly restricted. As natives tend to avoid the inferior segment, employers turn to immigrants to supply labour for these positions. This may affect the level of wages and create ethnic divisions. Notably, effects of immigration on recipient societies also apply to digital nomads, a phenomenon of economic migration that has emerged significantly in recent years, especially during the COVID‐19 pandemic. Unlike traditional migrants, these individuals relocate to different countries for remote work facilitated by technological advancements, such as artificial intelligence and social media. While their motivations often centre on lifestyle preferences rather than economic necessity, their movement reflects broader trends in economic migration shaped by globalisation and the changing nature of work (de Loryn, 2022). This trend can lead to increased property prices and gentrification, generating tensions in host communities, ultimately showing the complexities around human migration in today's age.

#### (c) Feedbacks, impacts, and models

The study of the consequences of human migrations and non‐native species introductions has led to the formalisation of feedback models that have shared conceptual mechanisms. The initial mechanism involves post‐establishment feedback, where migrants report back their success in new locations, potentially leading to a ‘bridgehead effect’ (Bertelsmeier & Keller, 2018) or relating to niche construction theory (Laland, Matthews & Feldman, 2016), where organisms actively modify their environment to enhance their survival and reproductive success, thereby influencing the selection pressures they face. Another relevant concept is ‘interspecific invasional meltdown’ (Simberloff & Von Holle, 1999), describing a process when immigrants create favourable conditions for subsequently arriving conspecifics (Liang *et al*., 2018). This concept, loosely comparable to the effect of ‘primary’ non‐native species lowering the resilience of invaded ecosystems to successive ‘secondary’ non‐native species introductions (Simberloff, 2006; O'Loughlin & Green, 2017), refers to an event where the initial success of a few individuals encourages further waves of migration, increasing the overall impact on the receiving environment. As migrants communicate their positive experiences, it can lead to an increased influx of newcomers, much as how a thriving non‐native species can pave the way for more individuals to establish (Simberloff, 2006). One key difference to note here is that whereas ‘invasion meltdown’ is fuelled by a general decrease of biotic quality, a ‘pioneering effect’ or ‘success feedback loop’ is, instead, mostly driven by the communication of successful positive experiences of first migrants within the new environment and not post‐establishment impacts.

Accordingly, human migration and the introduction of non‐native species can be shaped by socio‐economic factors and political conditions (e.g. access to affordable housing, language lessons, schooling, equal access to welfare for immigrant workers as for native workers), but also by cultural dynamics that influence migrants' integration into host societies (Dawson *et al*., 2017; Lenzner *et al*., 2022). Moreover, certain historical human migrations have resulted in significant ecological changes to ecosystems worldwide. These ecological disturbances have, in specific instances, affected the recipient environment, contributed to the decline of native species, and altered natural evolutionary processes, leading to long‐term impacts on biodiversity and ecosystem functionality (Diamond, 1997, 2011). As such, comparative attempts to quantify the effects of introduced non‐native species and human migrations exist. For non‐native species, the classic Parker–Lonsdale equation (Impact = Range × Abundance × Per Capita Effect) is often used to predict and describe their approximate impacts on ecosystems (Parker *et al*., 1999). This metric, but also others (Kumschick *et al*., 2024), help in assessing the potential threat of invasive non‐native species based on their distribution, population size, and individual impact. By contrast, known instances of historical human migrations that were devastating for the host society include those of early hominins (Bellwood, 2015) or during the Roman Empire (Money, 1976). As such, it is no surprise that the impacts of migrations today are evaluated as well, albeit through various multi‐dimensional and complex approaches. Economic models assess contributions to gross domestic product (GDP), employment, and fiscal balance, while demographic studies analyse changes in population structure and growth rates. Social integration indices, such as the Migrant Integration Policy Index (MIPEX), measure the success of migrants' assimilation into host societies (Huddleston *et al*., 2015). However, metrics like MIPEX have been critiqued for their potential biases (e.g. low reliability in individual dimensions) and challenges in accounting for cultural and contextual differences across countries (Ruedin, 2011; Niessen, 2014). These limitations underscore the need to supplement such metrics with qualitative insights to capture better the nuanced and dynamic realities of integration across diverse socio‐political contexts. Additionally, cultural impact assessments examine the influence on social cohesion and diversity, and policy impact analyses compare the effectiveness of different migration management strategies. These diverse metrics collectively provide a comprehensive framework for understanding and predicting the multifaceted impacts of human migration.

Cultural models offer diverse perspectives on the impact of migration on dominant societies. Models like Multiculturalism and Interculturalism emphasise the coexistence and mutual respect of diverse cultures, fostering cultural preservation and social cohesion through dialogue and exchange (Hugo, 2005; Werbner, 2005). Transculturalism and Acculturation highlight dynamic interactions and adaptation strategies, creating new cultural forms and hybrid identities. These models argue that migration can enrich dominant cultures by enhancing diversity and innovation. On the other hand, models like the Melting Pot and Assimilation model, as seen in contexts like the USA, suggest that migrants (should) adopt to the host country's culture and way of life. Additionally, certain models, such as the Cultural Convergence model, argue that migration can lead to a homogenisation of cultures (Rapoport, Sardoschau & Silve, 2018), analogous to the biotic homogenisation caused by the negative effects of non‐native species introductions on the presence and abundance of native species (Olden, Comte & Giam, 2018). These models propose that through processes like social mixing and cultural transmission, migration can generate a bilateral cultural convergence between home and host countries, leading to increased cultural similarity over time. Ultimately, whether through enrichment or convergence, migration can profoundly transform the cultural landscapes of societies (Bloemraad *et al*., 2023). Similarly, the global dominance of certain non‐native species, such as house sparrows (Anderson, 2006), parallels how cultural elements like Anglicisation have come to dominate globally, leading to a uniformity in cultural expressions across different regions (Kirvalidze, 2019).

System‐level and network theories, such as metacommunity theory, highlight the interconnectedness of populations across landscapes (see Bedford, 2005; Brown & Barney, 2021). Viewing these processes as complex adaptive systems suggests comparable features such as feedback loops, resilience, and tipping points, framing migration and invasion as adaptive responses to changing conditions. Both can be evaluated through cost–benefit frameworks, weighing economic, ecological, and cultural trade‐offs for both newcomers and host systems, while long‐term interactions often drive dynamic outcomes such as cultural exchange, hybridisation, or conflict, shaping co‐evolutionary trajectories in both human societies and ecosystems.

#### (d) Historical perceptions

Another moral similarity postulated in the past – following a broadly inclusive approach (Soulé, 1990) – is that neither human nor non‐human migration should be judged based on place of birth or a concept of nativeness. For humans, place of birth is a criterion, whose importance may be questioned on various grounds, for limiting freedom of movement (Ackerman, 1980; Carens, 1992; Kukathas, 2002). For other species, their non‐native status alone should not determine the priority for study, control, or management, which should be based on impact rather than origin (Davis *et al*., 2011; Schwindt *et al*., 2018). Moreover, in the view of acclimatisation societies in the 19th and 20th centuries on the introduction of non‐native species (Lockwood *et al*., 2013), the migration of humans has often been historically viewed as beneficial, particularly in the context of the USA, which embraced a self‐proclaimed philosophy of welcoming immigrants despite racial discrimination (Hing, 2004; Freeman & Kessler, 2008). While the integration of immigrants into host societies is ultimately reflected in tangible equal treatment and rights, the integration of non‐native species into the ecosystems where they are introduced is a much more relative and controversial scientific concept. It is considerably harder to determine insofar as it relies on a deep understanding of constantly evolving ecological concepts as diverse as the drivers of community assembly (Vellend, 2010), dynamic equilibria (DeAngelis & Waterhouse, 1987), and the meaning of biodiversity itself (Schlaepfer, 2018; Mammola *et al*., 2023). However, just as attitudes towards non‐native species shifted when perceived negative impacts became apparent, perceptions of immigration also changed in response to social and economic challenges, even though these perceptions of whether migrants are good or bad do not necessarily correspond to reality. The influx of diverse populations from various different backgrounds aiming to become ‘American’ (Archdeacon, 1984; Barone, 2013) contributed to the country's economic growth, cultural enrichment, and innovation, with 30 million Europeans having immigrated to the USA during the Age of Mass Migration (1850–1913; Freeman & Kessler, 2008; Palma, 2023). Yet, mass migrations can also pose substantial challenges if taken on naively. For instance, the partition of India in 1947 led to the largest mass migration in human history, with approximately 18 million people displaced (Bharadwaj, Khwaja & Mian, 2008). The sudden influx of refugees created immense social and economic strain, which was greatly influenced by the existing policies and social attitudes, resulting in widespread violence, resource shortages, and significant disruptions to both India and Pakistan (Bharadwaj, Khwaja & Mian, 2009). It is, therefore, no surprise that several authors have argued that any immigration policy must consider the potential economic, ethnic, and religious unrest caused by a large influx of immigrants (Kukathas, 2002). Similar care should be taken when addressing the introduction of non‐native species into new ecosystems. Policies tend to become restrictive when a large influx of immigrants is seen as disruptive to the functioning of a society, in order to protect the ongoing process of maintaining liberal values (Ackerman, 1980), as often discussed in terms of Muslim migration into secular majority‐Christian nations (Parekh, 2006; Hansen, 2011). This implies that a large influx from countries with different governance styles might prompt varying preferences for restrictions to preserve the state's cultural and societal (including governmental) norms. However, the impact of migration can be shaped positively through appropriate policies that leverage the economic, social, and cultural resources that migrants bring with them, thereby turning potential challenges into opportunities for growth and enrichment.

#### (e) Refugees and the introduction of non‐native species

The analogy between the relocation of human refugees and the introduction of at‐risk species, or some of their populations, for their protection or societal gains, is another compelling case, as both issues concern groups rather than individuals placed in specific locations (Ypi, 2008; Switzer & Angeli, 2016). Non‐native species are introduced either: (*i*) accidentally following an escape; or (*ii*) intentionally for a specific benefit, function, or service (Pyšek, Jarošík & Pergl, 2011) or even as a symbol of status and prestige [e.g. wildlife trafficking as luxury goods in black markets (Dawson *et al*., 2017; Bortolus & Schwindt, 2022)]. Considering the rising number of native species extinctions due to anthropogenic factors, endangered species represent a major conservation challenge (Alfonzetti *et al*., 2020; Senior *et al*., 2024). In some cases (i.e. rewilding initiatives, assisted migration in response to climate change), these species require management such as assisted translocations (Moritz, 2004; Donlan *et al*., 2006; Mueller & Hellmann, 2008), which may involve the intentional relocation of species outside their historical biogeographic ranges (i.e. those they have a history of co‐evolution with) to prevent species loss (Hoegh‐Guldberg *et al*., 2008). In their new environment, these species can establish (i.e. thrive, adapt, and move), or go extinct in response to the local conditions or future environmental changes (Minteer & Collins, 2010). For example, the Guam kingfisher *Todiramphus cinnamominus* became extinct in the wild due to predation by the brown tree snake *Boiga irregularis* introduced in the 1940s (Clark, Clark & Siers, 2017). Conservation efforts are underway to reintroduce the species to Palmyra Atoll as part of a captive breeding programme aimed at eventually restoring their population on Guam (Trask *et al*., 2021). Thus, animal and plant species may sometimes be translocated because they are threatened in parts of their native range or, which is more often the case, to be used and possibly exploited in a new area for economic and other reasons, such as commodities (Bowling, 1942). Introducing species to new environments, however, requires deep *a priori* consideration (Guareschi *et al*., 2024), and carries the risk of them becoming invasive, harming native species, and disrupting the recipient ecosystem and its functioning (Tarkan *et al*., 2024). However, preserving species solely in captivity can become a larger ethical burden and decouples these from selective pressures in their habitats (especially if functionally extinct), suggesting that unconventional strategies may be necessary for their preservation (Minteer & Collins, 2010; Conde *et al*., 2011).

Similarly, theories of human migration emphasise special obligations towards humans at elevated risk in their home country, recognising refugees as a special group needing protection (Arendt, 1967). These refugees (as a specific legal category) are often forced to leave their country of origin (*i*) due to extreme safety risks (e.g. violent conflicts, political persecution, severe human rights violations; Seglow, 2005), (*ii*) when political entities use human migrations as a means (i.e. by weaponizing migrants as so called ‘foreign policy bargaining chips’; Greenhill, 2016) to, for instance, destabilise regions or exert political pressure (Kotoulas & Pusztai, 2020), or (*iii*) to escape natural disasters (Biermann & Boas, 2008). The current situation regarding the migration of refugees – from an economic standpoint – —can only be handled efficiently through closer international cooperation in the field of aid and asylum policy. Despite potential criticism, recent mass migrations have often led to an uneven distribution of refugees within larger political entities like the European Union, which is perceived as unfair (Altemeyer‐Bartscher *et al*., 2016; Holtug, 2018). This imbalance can hinder the overarching goals of protecting refugees and attaining successful integration (Maldini & Takahashi, 2017). Moreover, while this highlights the complexity and challenges in creating a perfect theory of species migration (Ypi, 2008), statelessness (https://www.state.gov/other-policy-issues/statelessness/) deprives individuals of rights and threatens global stability (Switzer & Angeli, 2016). This situation is conceptually similar to how the extinction of endangered species challenges conservation efforts, demonstrating that assisted colonisation is not a viable conservation strategy (Ricciardi & Simberloff, 2009), whereas addressing its causes or any threat at its roots is.

### (4) Contrasts and distinctive differences

There are numerous striking parallels between the introduction of non‐native species and human migrations, yet these parallels must be understood with nuance and a clear recognition of the distinct underlying dimensions, values, and contexts involved in each to avoid harmful and erroneous generalisations. Moreover, notable opportunities but also substantial challenges can be presented by both non‐native species introductions (Simberloff *et al*., 2012, 2024) and human migration (Benitez, 2004). As such, these visible parallels in introduction, establishment, spread, and impact make it easy to draw analogies between the two processes, suggesting a (superficial) similarity in the dynamics of introduced non‐native species and human migration. Yet, even within specific cases of biological invasions in different contexts, there remain notable differences between introduced non‐native species in terms of achieving a successful establishment process, population growth, spread, and exerted impacts at the population level (Haubrock *et al*., 2024), which may be seen as analogous to differences in the successful integration of human migrants from distinct socio‐cultural and ethnic backgrounds in recipient societies. Indeed, despite the aforementioned similarities, there are fundamental differences between biological invasions and human migration that stem from their distinct underlying mechanisms, impacts, and intrinsic values. Intuitively, four key differences between human migration and introductions of non‐native species become obvious.

#### (a) Rights and ethical considerations

Human migration encompasses a complex interplay of socio‐economic, political, and humanitarian factors (Black *et al*., 2011; Pemunta & Aristide, 2013; Lupak *et al*., 2022). Unlike non‐human species, humans are often argued to possess ‘different’ rights, cultures, and moral considerations that necessitate a different framework when discussing non‐native species introductions *versus* (large‐scale) human migration (Caviola *et al*., 2022; Yen & Cheong, 2023). This viewpoint can become complex when distinguishing native from non‐native species or when discussing non‐native species posing considerable ecological and economic threats in their introduced range, while being threatened in their native area (Copp *et al*., 2005). In contrast to ethical considerations for non‐native species, immigration policies involve both individual and group rights. The immigration controversy arises from how states variably recognise and grant access, reception, recognition, and citizenship rights to non‐citizens, based on national laws and international conventions. Human immigration thus hinges on migratory regimes established between states and regions, determining the conditions and consequences for those crossing borders. Human rights must be recognised by states, which varies significantly depending on one's passport and whether states have signed and respect international conventions granting such rights to all humans. Conversely, in non‐human species conservation, currently, the focus is on genes, populations, species, and ecosystems, rather than individuals. Nevertheless, the pain and suffering of individual animals are increasingly acknowledged, as evidenced by changes to control and eradication measures in several countries, including the banning of anticoagulant rodenticides and recent calls for reducing the use of lethal methods to study both vertebrates and invertebrates (Lillywhite *et al*., 2017; Lövei & Ferrante, 2024). This new awareness culminated in the signing of the New York Declaration on Animal Consciousness on 19 April 2024 (https://sites.google.com/nyu.edu/nydeclaration/declaration), which also includes evidence of invertebrate sentience.

#### (b) Conscious reasons for movement

Human migration typically occurs because individuals choose actively to move for personal reasons, except in cases where refugees are forced to flee. By contrast, while native species may move naturally following changing environmental conditions (Lenoir & Svenning, 2015), and then are not considered invasive (defined by the occurrence of active or passive spread) as this pertains only to non‐native species introduced outside their native area (Soto *et al*., 2024a), the introduction of non‐native species to new areas is by definition due to human actions. However, secondary introductions (i.e. the further translocation of individuals away from their primary introduction site – bridgehead effects) can occur either naturally or as a result of human facilitation (Bertelsmeier & Keller, 2018). Thus, a fundamental difference is that contemporary biological invasions are fundamentally human‐mediated, and therefore closely track patterns of human movement and their goods (Dawson *et al*., 2017; Lenzner *et al*., 2022; Andrés *et al*., 2023). Human migration, however, is, but only to some extent, an autonomous and conscious decision, considering the aforementioned relevance of external forces (Switzer & Angeli, 2016). This means that the migration–invasion influence is more unilateral when considering potential drivers, and thus, non‐native species introductions are not driving migration, but are a symptom of it.

Unlike non‐native species, human migrants nowadays often have access to detailed, yet incomplete or sometimes misleading, information about their destination, including climate, economic conditions, legal frameworks, potential risks and benefits, and cultural acceptance (Felton, 2015; Kluge *et al*., 2020; Yarwood *et al*., 2022). Theoretically, this wealth of information allows migrants to make informed decisions and strategically plan their relocation, systematically increasing their likelihood of successful integration and adaptation to the new environment. By contrast, non‐human species generally have no inherent mechanisms to assess or respond to the conditions of their new habitats aside from their natural response [e.g. behavioural changes, ecological and eco‐physiological adaptations, range shifts (Richards *et al*., 2006; Hôrková & Kováč, 2014)]. This often results in a high rate of unsuccessful invasions, as the introduced species may not survive or thrive due to unsuitable environmental conditions [e.g. lack of necessary seasonal adaptations, competition with native or other non‐native species, or other ecological factors (Kowarik, 1995; Musolin & Numata, 2003; Zenni & Nuñez, 2013)]. Moreover, human migrants can make informed decisions to move away from unfavourable environments, or towards better opportunities, based on their access to information about potential destinations. This proactive approach allows them to seek out regions with favourable economic and climatic conditions, social acceptance, and legal frameworks that support their long‐term well‐being and success. While unfavourable (e.g. cultural and socioeconomic) conditions may limit their establishment, or increasingly suitable conditions may facilitate their range expansions (Matlin *et al*., 2018; Lebano *et al*., 2020; Kanengoni‐Nyatara *et al*., 2024), these human movements are driven by external factors rather than deliberate choices.

#### (c) Scale and impact

The scale and numbers needed to impact the receiving community differ between human migration and non‐native species introductions (Switzer & Angeli, 2016). Even advocates of open borders agree on stricter immigration policies if large numbers (i.e. a high propagule pressure) would negatively affect the state, but they do not believe that a small number of immigrants could destabilise a society (see Hansen, 2011; Maher, Aljafari & Maher, 2017). By contrast, even a small population of an introduced non‐native species, particularly those with *r* reproductive strategies (i.e. rapid developmental rate and high fecundity; Casties & Briski, 2019), can significantly affect an ecosystem by rapidly growing in abundance, threatening native species and upsetting the existing ecological balance, with hard‐to‐reverse impacts even shortly after being introduced (Duncan & Forsyth, 2006; Everts *et al*., 2024). In other words, biological invasions are generally viewed as a negative process (despite sometimes being viewed by citizens as beneficial or worth protecting; Crowley, Hinchliffe & McDonald, 2017), while human migration is often politically portrayed as beneficial, especially in terms of economic and cultural contributions (Yen & Cheong, 2023), largely motivated by a feeling of hope and the expectation of welfare improvement. Although this is not always the case (Scholten, 2022), rising voices in political discourse increasingly contest this viewpoint, reflecting a growing divide in public perception (Arcimaviciene & Baglama, 2018). This is reflected in the relatively higher success rates of human migration compared to non‐native species invasions, where informed decisions and pre‐arrival preparations, as well as conditions in recipient societies, facilitate smoother transitions (Johnson & Baumal, 2016; Norberg, 2017; Bhuyan & Schmidt, 2019). However, if individuals or groups of individuals migrating to a new area are subject to poor conditions upon arrival [as in the Mediterranean since 1960 (de Haas, 2011; Fargues, 2017; McMahon & Sigona, 2018)] where migrants are subjected to unsafe conditions and excessive deaths, then the difficulty of integration in the receiving country may increase (Norwegian Ministries, 2022; Phillimore *et al*., 2023).

The impacts of biological invasions and human migration also differ in scale and permanence. While invasive non‐native species can irreversibly alter ecosystems and threaten biodiversity, the success of non‐native species depends on the species' invasiveness (Ricciardi & Cohen, 2007; Catford *et al*., 2016), including propagule pressure and genetic diversity of introduced populations as well as ecosystem‐specific factors like environmental filtering, biotic resistance, and ecological niche availability, which define an ecosystem's invasibility (Alpert, Bone & Holzapfel, 2000; Kühn & Klotz, 2007). Unlike non‐native species that may or may not be perceived as negative or positive based on human values, human migrants tend actively and consciously to seek integration, employment, and social acceptance, often contributing to diverse cultural landscapes and economic growth, e.g. by fostering economic innovation and flexibility (Peri, 2012). By filling key labour gaps and introducing new cultural perspectives, they can significantly contribute to the dynamism of the host economies. As such, human migration's impacts on host societies can vary widely based on policy responses, socio‐cultural assimilation, and economic integration. This means that much of the influence of migration (and how it is perceived) comes down to perception; even a trickle of a certain group of migrants can be framed as terrifying and used to enact draconian policies. A key difference is also human agency: it is up to the receiving environment how human migration is conceptualised and managed. Migration can be considered beneficial or detrimental based on perceptions, values, and actions (Maher *et al*., 2017). A society can view it as a positive force and invest resources to welcome migrants and utilise their potential to serve society – or not. The successful integration of humans into local societies, however, differs significantly from the successful integration of non‐native plants or animal species into a new environment. This is primarily because the concept of an environment for humans – a ‘society’ or ‘community’, defined socially and historically and governed by a state – differs greatly from what constitutes an environment for non‐human organisms [i.e. the prevailing abiotic and biotic conditions (Abrams, 1982; Robbins, Hintz & Moore, 2022)].

While individual cases of non‐native species introductions that are retrospectively highly valued for their ecosystem services or functions exist (Shackleton *et al*., 2020; Kelsch *et al*., 2020), successful integration of migrants most often leads to societal enrichment through diversity, innovation, and labour contributions, whereas poorly managed migration can strain social services, exacerbate cultural tensions, and fuel political divisions. For example, cities in Western Europe with proactive integration policies have often experienced substantial economic growth and cultural enrichment, particularly when these policies were implemented in the post‐World War II era – a period marked by significant movement of populations (Vonyó, 2018). Migrants contributed to innovation and expanded the labour markets, under the Marshall Plan's provision of crucial financial assistance that helped rebuild West Germany's infrastructure and industry (Payne, 2011b, Payne, 2011a). Major urban centres in Germany benefited from a diversity of talents and ideas that fostered economic dynamism (Hooper, Desiderio & Salant, 2017) through policies that helped in the assimilation of migrants' lives, ranging from social welfare programmes to employment opportunities, and leveraged the skills and talents of the migrants (Shaev & Hackett, 2021).

#### (d) The cosmopolitan nature of a connected humanity

Unlike non‐native species which are defined by their introduction to environments that were naturally unreachable mainly due to geographical barriers, *Homo sapiens* has been a cosmopolitan species (i.e. with a global distribution) for more than 12,000 years (Potts, 2013). While acknowledging significant ethno‐cultural differences, humans have historically adapted to and thrived in diverse environments across the globe (Smithers & Smit, 1997; Kuzawa & Thayer, 2011; Scheinfeldt & Tishkoff, 2013). Throughout history, human societies have demonstrated remarkable resilience and flexibility in the face of environmental changes, migrating across continents and establishing complex civilisations. Despite the diverse cultures and languages, humans have become increasingly connected, sharing knowledge, innovations, and cultural practices through trade routes, exploration, and now digital communication (Kirby *et al*., 2016). This interconnectedness has fostered a global community where all sorts of goods, services, ideas, and technologies are exchanged at unprecedented rates across terrestrial, aerial, and aquatic routes. Accordingly, a fundamental difference between the introduction of non‐native species and large‐scale human migration lies in the inherent interconnectedness and the increasingly sophisticated and instantaneous communication and information mechanisms available to humans (Poot, 1996; Riva, 2005).

### (5) Safeguarding mitigation

State policies govern migration processes – they do not just happen organically. One of the reasons why politicians have occasionally linked non‐native species to human migrations has been the superficially similar role of national barriers to that of geographical barriers limiting non‐human species distributions (Okólski & Salt, 2014). When conceptualising ‘the border’, we are actually discussing migratory regimes established by states, which determine the mechanisms of immigration, the crossing of borders, and the subsequent integration of immigrants. While barriers to non‐human species distributions are features of the biophysical world shaping the biogeography of species, the concept of political borders has increasingly fuelled tension between factions with opposing views on human migration. Borders, while primarily seen as barriers, not only change over time but also function as zones of interaction where cultural and economic exchanges and cooperation can occur. These interactions often lead to more nuanced understandings and shared policies between nations. As such, borders can function as both barriers and bridges (Donnan & Wilson, 1999). In the field of invasion science and policy, borders are crucial for protecting biodiversity and economic interests, by preventing the introduction of non‐native species (as they allow for the regulation and inspection of goods and organisms crossing into new locations; Hulme, 2013). Conversely, the historically important barriers for species were eco‐evolutionary ones (Cano‐Barbacil, Radinger & García‐Berthou, 2022). Barriers to non‐human species thus differ significantly from those relevant for humans (Tarkan *et al*., 2024), although physical human‐made borders (e.g. walls and dams) are becoming increasingly important as eco‐evolutionary forces (Dallimer & Strange, 2015; Peters *et al*., 2018; Liu *et al*., 2020). Their effectiveness may, however, depend on several factors, including scrutiny of their enforcement, control, and, ultimately, the country's respective size (Hulme, 2013). In human migration, unlike ecological contexts, borders raise significant ethical and humanitarian issues by simultaneously protecting and violating human rights, offering the critical function of governing human movement (Betts, 2013).

Borders do, however, illustrate the complex considerations that distinguish the introduction of non‐native species from human migration (see e.g. Switzer & Angeli, 2016). From a liberal egalitarian point of view (Carens, 1992), borders are often claimed to create humanitarian and economic issues by restricting free movement, separating families and limiting opportunities for those seeking better lives (Carens, 2008; Switzer & Angeli, 2016). They exacerbate inequality, trap people in regions with limited resources, and/or prevent efficient labour allocation (Hass, 2014). Strict enforcement can presumably lead to human rights abuses and international tensions over territorial disputes (Carr, 2015). National borders also delineate states and their primary interest, which may hinder global or large‐scale cooperation (by e.g. larger political entities like the European Union) on issues like climate change, public health, and trade, while reinforcing divisions and impeding cultural exchange and perpetuating global inequities and social fragmentation (Helliwell, 2000). National borders are, however, simultaneously essential for maintaining national security, sovereignty, and economic stability by controlling who and what enters and exits a country, thereby protecting against external threats and managing immigration (Atzili, 2011; Tazzioli, 2019). They may structure cultural identities (being built on shared values, traditions, and collective experience that give a group of people a sense of identity and belonging), enforce laws, and facilitate resource management, while also contributing to public health by controlling disease spread. Borders are also argued to support political stability, may reduce territorial disputes, and enable economic protectionism to safeguard domestic industries, collectively ensuring a nation's ability to govern itself effectively and sustainably (Blake & Gilman, 2024).

Biosecurity for non‐native species and efforts to mitigate illegal human migration thus share certain similarities in their focus on border control and movement management. Yet, they diverge significantly in their underlying objectives, methodologies, and broader impacts. Both are concerned with regulating movement across national borders. Biosecurity aims to safeguard ecosystems, agriculture, and public health from non‐native species and diseases through monitoring, risk assessment, quarantine measures, and stringent regulations applied to goods, cargo, and travellers at places of entry (Meyerson, Meyerson & Reaser, 2009; Singh, Ash & Hodda, 2015; Lieurance *et al*., 2023). Similarly, mitigation of illegal human migration involves managing unauthorised entry and movement of individuals outside legal frameworks, employing strategies such as law enforcement, border patrols, immigration policies, international agreements, and humanitarian aid efforts (Grant & Solicitors, 2005; Pickering *et al*., 2016). However, their objectives differ slightly: biosecurity prioritises ecological preservation and safeguarding public health (Meyerson *et al*., 2009; Gorgile, 2023), while illegal human migration management focuses on security risks, economic impacts, humanitarian concerns like human rights abuses, and integration challenges in host societies (Bogusz *et al*., 2004; Grant & Solicitors, 2005). The methods employed reflect these divergent goals, with biosecurity relying on scientific expertise and quarantine protocols [which also existed for humans until recently (Conti, 2008; Cetron, 2016)], and migration control utilising enforcement measures and international agreements (Champion, 2018).

Consequently, their effects also vary: biosecurity measures can influence trade and international relations (Serban, 2021), whereas efforts to stop illegal migration poses broader socio‐economic, political, and humanitarian challenges, impacting labour markets, cultural integration, and international cooperation efforts (Donato & Massey, 2016; Christiaensen, Gonzalez & Robalino, 2019; Fasani, Llull & Tealdi, 2020). Thus, while both fields intersect in border management, their distinct purposes, strategies, and consequences highlight the multifaceted complexities of managing cross‐border movements in today's increasingly interconnected world. Areas with restrictive migration policies, especially in rural regions or during periods of economic downturns, saw increased social tensions and economic stagnation. This dichotomy underscores how the management of migration can either strain social services, exacerbate cultural tensions, and fuel political divisions, or contribute to a more dynamic and cohesive society. Such outcomes are heavily influenced by the historical, political, and cultural contexts of each region, as well as by the scale of migrant inflow (Ottaviano & Peri, 2012, 2013). Here lies an appreciable difference with the management of non‐native species introduction. Even positive effects can be delineated, few policies have succeeded in taking advantage of successful non‐native species (e.g. the intensive farming of wheat as food resources). Therefore, while both biological invasions and human migration involve the introduction and establishment of newcomers influenced by human actions, their outcomes and management require distinct approaches tailored to their respective ecological or societal contexts. Understanding these differences is crucial for developing effective strategies to mitigate negative impacts and harness potential benefits from both phenomena.

## III. WHY COMPARISONS CAN BE DANGEROUS

Despite the surface‐level similarities, equating the introduction of non‐native species with human migration may be argued to be fundamentally flawed and dangerous and so these two phenomena should not be directly compared. The distinct ethical, social, historical, biological, and humanitarian contexts that characterise each make such analogies obsolete, weak, and inappropriate from a scientific point of view. Trade and travel, the expansion of empires, colonialism, and globalisation have historically played a significant role in shaping the processes and outcomes of both non‐native species introductions and human migrations (Crosby, 2004). Unlike humans, non‐native species populations (currently) do not have rights or moral considerations (Francione & Charlton, 2017; Riley, 2019), except for specific cases involving the protection of natural reserves, the ethical regulation of scientific experiments, or euthanasia; however, some activists are advocating for the attribution of rights to non‐native species (Perry, 2004). Although recent research highlights differing views on the positive and negative impacts of non‐native species (Schlaepfer, 2017, 2018; Sax *et al*., 2022; Lockwood *et al*., 2023), negative effects are often seen in environmental biodiversity loss and human livelihoods. This is reflected in the rising economic costs of introduced species (Diagne *et al*., 2021; Roy *et al*., 2024). Some studies, however, acknowledge the positive effects of certain non‐native species on ecosystems, leading to frameworks that recognise these benefits (EICAT+; Vimercati *et al*., 2022). By contrast, human migration is a socio‐political issue involving individuals with rights and cultural diversity. Migration is often driven by humanitarian needs, such as escaping inhumane conditions and conflicts, including proxy wars (Popescu, 2016; Patterson, 2018; Castelli, 2018). However, migrants also move for other non‐altruistic reasons, including the desire for better economic opportunities, even when displaced by conflict (Benson & O'Reilly, 2009). The effects of these migrations on recipient societies and cultures, but also on the countries they originate from (i.e. the loss of qualified personnel; Iredale, 2001), are multifaceted and difficult to describe, requiring nuanced and differentiated responses from support systems that are tailored to human well‐being. By contrast, addressing the effects of introduced non‐native species involves rigorous biosecurity and the application of clear‐cut, often pre‐determined, management actions.

Nonetheless, some politicians and media exploit superficial similarities between the introduction of non‐native species and human migration as a scapegoat for existing issues and to distract from the root drivers of migration. Specifically, this rhetoric often overlooks the historical and legacy effects of e.g. colonialism (Gutiérrez Rodríguez, 2018), which have left many regions economically and politically destabilised, and the unequal global distribution of wealth, which largely drives current human migration flows by functioning as a pull factor (Bertocchi & Dimico, 2019; Ferwerda & Gest, 2021). Despite this background, inadequate efforts to support the Global South further exacerbate these issues, as many countries in these regions continue to struggle with poverty, lack of infrastructure, and limited opportunities. Moreover, global environmental change, with its deleterious effects on human livelihoods, further compounds the challenges faced by these regions. Conflating the challenges of human movements with those of non‐native species ignores the inherent human drive for prosperity and safety, and our instinct for exploration, expansion, and conquest. Additionally, the rise of authoritarian, corrupt, and ineffective political systems is another prime cause of migration, as individuals seek refuge from oppressive regimes and unstable governments (Dobrovidova, 2023). This comparison further fails to recognise the agency and dignity of migrants, who often move in search of better lives, safety, and opportunities for themselves and their families. Politicians who draw these comparisons may use them to justify xenophobic policies and rhetoric, portraying migrants as a threat to national identity, security, and resources. This not only fosters discrimination and social division but also diverts attention from the systemic issues that need addressing, such as global inequality and the legacies of colonial exploitation. Although debatable (see e.g. Regan, 1983), populations of non‐native species are currently not recognised as having the same legal rights as populations of native species as they are managed and valued differently (Schlaepfer, 2018; Stevenson *et al*., 2023). Human migrants, however, do possess inherent group and individual rights and the movement of people has historically contributed to cultural exchange, economic growth, and societal development (Ratha, Mohapatra & Scheja, 2011; Nathan, 2014), whereas non‐native species introductions more often than not disrupt ecosystems and biodiversity (Charles & Dukes, 2007; IPBES, 2023). Moreover, such comparisons can obscure the responsibilities of wealthier nations in both creating and addressing migration issues. These countries often benefit from the global economic system that perpetuates inequality and are sometimes directly involved in conflicts or policies that displace people. Addressing migration requires a nuanced understanding of these dynamics and a commitment to global cooperation, equitable development, and humanitarian assistance (Bertocchi, 2016; Gutiérrez Rodríguez, 2018).

Comparing non‐native species introductions to human migration is also problematic because it ignores the fundamental rights and agency that humans possess, a consideration that is not typically extended to non‐native species. This contrast highlights the ethical difference in how we view human migrants *versus* populations of non‐human (especially non‐native) species. Drawing parallels with populations of invasive non‐native species undermines these contributions and perpetuates harmful stereotypes (Bernstein, 2015; Howard, 2019; Inglis, 2020). Rather than viewing migration solely through a lens of *impacts*, policies must recognise the ethical imperative to uphold human dignity and address the systemic factors driving migration. Effective governance should prioritise inclusive integration strategies that foster mutual understanding and cooperation among diverse communities. By reframing the migration discourse away from reductionist biological analogies towards a holistic socio‐political perspective, policymakers can better address the complexities of migration while promoting global solidarity and sustainable development (Laine, 2020; Dennison, 2021).

Equating human migrants with non‐native or even invasive non‐native species can subsequently lead to dehumanising rhetoric and policies, concomitantly fostering xenophobia and discrimination. Such comparisons can oversimplify the complexities of human experiences and ignore the socio‐economic and political contexts that drive migration. Furthermore, this analogy can perpetuate harmful stereotypes and justify restrictive or punitive measures against migrants, undermining human rights and social cohesion. It also risks diverting attention from the real challenges and opportunities of migration, such as addressing the root causes of displacement and investing in effective integration strategies. Therefore, while the processes of introduction, spread, and impact might superficially seem similar, the underlying ethical and social implications are vastly different, making the direct and generalising application of invasion biology principles to human migration not only inappropriate but also potentially dangerous.

Key differences and synergies between human migration and non‐native species introductions can be outlined by highlighting five key aspects:

(1) *Transport*: the origins and destinations of immigrants today differ from the past and are driven by contemporary geopolitical and economic dynamics, whereas non‐native species transport is influenced by trade and travel networks. Historically, migration follows external drivers and patterns, whereas non‐native species introductions often result from unilateral trade practices, leading to different sources and destinations for both.
(2) *Establishment*: intentional introductions of plants and animals, as well as formal immigration, require rigorous screening based on potential risks and costs to the recipient nation. Illegal imports and immigrants are managed similarly through profiling and pathway analysis, and there appears to be a correlation where countries with strong biosecurity systems also maintain stringent immigration controls.
(3) *Spread*: facilitated by human activities, non‐native species often spread within recipient regions, while evidence suggests that immigrants tend to remain in urban areas, with limited mobility to remote districts unless incentivised or required by visa conditions. This difference highlights the disparate spread patterns of non‐native species and human migrants.
(4) *Impact*: the impacts of non‐native species and human migration differ significantly, although both are influenced by value systems and perceptions. Non‐native species can disrupt ecosystems and threaten biodiversity, while human migration impacts are multifaceted, potentially influencing economic growth, cultural diversity, and social dynamics.
(5) *Synergy*: since invasive non‐native species can often be considered remnants of past human (direct or indirect) activities, the study of biological invasions has significantly enhanced our understanding of the global spread of humans and their cultures. We have gained deeper insights into human dispersal patterns by examining species introduction routes. For example, the presence of non‐native domesticated plants, animals, and their associated species (including invertebrates and infectious microorganisms) has enabled archaeologists to trace the arrival of agricultural practices in various regions worldwide. Similarly, understanding the pathways, vectors, and causes of human dispersal not only aids in predicting future species introductions but also helps uncover past events that have yet to be fully recognised.

## IV. GOING FORWARD

There are arguably numerous ethical implications of applying invasion biology concepts to human migration, resulting in an oversimplification and misrepresentation of the complex socio‐political dynamics of human movements (Vilà *et al*., 2021; Bortolus & Schwindt, 2022). Hence, works like Pitoski *et al*. (2021) excluded studies on animal movements when reviewing factors of human migration, just like major works on non‐human migrations (e.g. Sauer, 1988; Dingle, 2006, 2014; Nathan, 2008) tend to exclude human movements (Faulkner, Hulme & Wilson, 2024). In other examples, the use of agent‐based modelling to study migration policies has been explored, reflecting the emergent properties seen in biological invasions (De Luca *et al*., 2022). Research has, however, shown how human movements can facilitate the spread of non‐native species (Lenzner *et al*., 2022; Andrés *et al*., 2023), including pathogens causing disease (e.g. McNeill, 2004) and, conversely, how invasive non‐native species can affect human populations by acting as vectors for diseases (Vilà *et al*., 2021). Similarly, through the study of historical ecology and species introduction pathways, experts were able to reconstruct the behaviours, migration routes, and trade networks of ancient civilisations (Hofman & Rick, 2018). This interplay between biological invasions and the movement of humans and human health provides a concrete example of how invasion biology concepts can be relevant to human activities and highlights the potential to integrate social science perspectives into invasion biology. This includes the study of human dimensions underlying biological invasions, such as the social, economic, and cultural impacts of invasive non‐native species, and *vice versa*, exploring how invasion biology concepts could illuminate aspects of human migration (Vilà *et al*., 2021; Bortolus & Schwindt, 2022).

To avoid deleterious comparisons between biological invasions and human migrations, one must navigate the complexities without succumbing to oversimplification. While both biological invasions and human migration involve the movement of organisms or populations across geographical boundaries and both are influenced by social, economic, political, and historical factors, their dynamics and global patterns are fundamentally distinct (Gold, 2000; Stark & Wang, 2002; Chapman *et al*., 2017; Bernery *et al*., 2024). However, invasion scientists seldom focus their research on human migrations alone. Concomitantly, social scientists, who are experts in human migrations, do not concentrate their research on non‐human species. This is a fundamental intrinsic disciplinary partitioning of interests that strengthens the value of multidisciplinary collaborations. It is, therefore, imperative for researchers from various disciplines to approach these topics with rigorous scientific scrutiny and avoid the temptation to generalise or politicise their findings. Striving for objectivity allows scientists to uncover the nuances that distinguish these phenomena and their impacts on ecosystems and societies. This effort to remove bias is essential for producing trustworthy research that informs policy and public understanding without being swayed by ideological agendas (Russell & Blackburn, 2017). Disciplines such as Sociology, Anthropology, Post‐colonial Studies, and Critical Theory widely acknowledge that concepts and methods are largely influenced by those who use them and their social positions, often of advantage and superiority (Gergen, 1992; Hammond & Wellington, 2012; Susen, 2020). Terms like ‘beneficial’ or ‘integrated’ in the context of immigration and immigrants are not objective facts but value statements. They subjectively depend on an idea of desired traits that immigrants must have to be deemed suitable for inclusion in the national body, or on whether the government at a given moment decides that immigration of skilled workers will contribute a net positive to the economy (Chang, 1997; Maher *et al*., 2017). While recognising that scientists, like all individuals, have their own values and biases, adhering to empirical evidence, methodological rigour, and the scientific method can provide valuable insights that transcend political divides and contribute to informed decision‐making. The risk of politicisation in scientific discourse threatens the credibility of research outcomes (Everson & Vos, 2009; Weitkamp *et al*., 2024). When scientific inquiry is influenced by political agendas, there is a danger of cherry‐picking data or interpretations to fit preconceived narratives, undermining the integrity of the scientific process. Therefore, while scientists should be aware of and actively engage in the application of their work to better the world, they must also strive to maintain neutrality in their research methods to preserve the integrity of their findings (Rotblat, 1999).

In understanding invasion rhetoric and the associated narratives (Banulescu‐Bogdan, Malka & Culbertson, 2021; Soto *et al*., 2023), it becomes essential to clarify that invasion science terminology should not be co‐opted by activists, journalists, and especially politicians, as it is essential to maintain the integrity and neutrality necessary to benefit society through informed governance and policymaking. However, we argue that it is important to be cautious about terminology. As was common practice in the field of Ecology during the second half of the 20th century (McIntosh, 1995) following two World Wars, militaristic language is also found in widely accepted theories among invasion scientists, including the ‘invasional meltdown’, ‘enemy release hypothesis’, or the ‘novel weapon’ theory (Daly *et al*., 2023). Moreover, the use of unclear terminology or its misuse by some activists, journalists, and politicians can cause significant misunderstandings among the public, leading to misplaced fear, misguided policies, and the stigmatisation of both species and peoples (Frank, 2021). Regarding the comparison of invasion science, particularly the phenomenon of non‐native species introductions, with migrations, it becomes essential to distinguish between the bureaucratic or legal language of the state, the scientific language, and the rhetoric used by politicians. Bureaucratic language, such as referring to foreign citizens as ‘aliens’, reflects older terminology that is now being gradually revised to remove historical injustices and discrimination but is still being used as such by some politicians. Scientific language, especially in the social sciences, has been carefully and thoughtfully revised to avoid terms like ‘invasion’ or ‘aliens’, which are now only used exceptionally and to the disrepute of scholars who employ them (Matvieieva & Matvieiev, 2023). By contrast, politically charged language often scapegoats immigrants for social problems, thus exemplifying how different forms of language produce distinct effects within their social fields, influencing public perception and policy in various ways. In this context, invasion science could learn from the social sciences, as examples from fields such as sociology, urban studies, and migration studies – where the language of invasion science was used a century ago – have long been denounced for their inappropriate language and have since evolved to adopt more accurate terminology and contextualisation. For instance, the Chicago School of Sociology in the early 20th century often used terms like ‘invasion’ to describe the movement of immigrant populations, which has since been criticised and replaced with language that recognises the complex social dynamics and contributions of immigrants (Cavan, 1983; Coates, 2007). This means that particular attention has to be given to the terms used in invasion science and emphasises the importance of using language that accurately reflects the complexities of both biological invasions [see Soto *et al*. (2024a) for further discussion] and human migrations. Additionally, knowledge brokers should be more careful in how they search for and translate evidence from non‐English publications, ensuring that terms like ‘invasive’, ‘invasion’, ‘invader’, ‘alien’, and ‘colonisation’ are accurately and appropriately rendered in English when advising policymakers in international contexts.

## V. IMPLICATIONS

While this review already addresses significant aspects of human migration, it is crucial to consider that foreseeing the future remains impossible, including future wars that cause migration waves or the varying effects of climate change on future migration patterns. Similarly, invasion science can only aim to identify patterns and trends that lead to non‐native species becoming invasive and exerting significant impacts (Soto *et al*., 2024b). While there seem to be some superficial similarities in the processes underlying biological invasions and large‐scale human migration, the distinct ethical, social, and biological dimensions require careful, context‐specific analysis and policies tailored to the unique challenges and opportunities that each present. Non‐native species are not only introduced by humans; humans also define what is considered beneficial or harmful, and what constitutes ecological equilibrium, based on human goals, values, and specific conceptualisations of ‘nature’. While xenophobic attitudes towards non‐native species can reinforce negative views of minorities, protecting native biological communities is a legitimate and necessary conservation goal (Heppes & McFadden, 1987). The *Convention on International Trade in Endangered Species of Wild Fauna and Flora* (Heppes & McFadden, 1987), the Endangered Species Act (1973), and international biodiversity agreements such as the *Kumming‐Montreal Global Biodiversity Framework* and the *Convention on Biological Diversity* (CBD) imply that certain taxa belong to specific areas. Although biological diversity changes over time (Terborgh, 1974), pride in a region's natural heritage is valid (Escobar, 1998) and aligns with arguments supporting Indigenous peoples and local communities, as well as their culture, language, and intrinsic relationships (Jordan III, 1994; Hettinger, 2001; Roy *et al*., 2024). Protecting native species and native ethnic groups is essential for maintaining ecological balance and cultural integrity (Bond *et al*., 2019; Serrano‐Rojas *et al*., 2022). Studying and discussing non‐native species introductions and human migrations, therefore, requires a nuanced approach. Without nuance, there is a risk of oversimplifying complex issues that may lead to dehumanisation or governance that ignores the risks associated with uncontrolled migration. Moreover, while a blanket argument against the introduction of non‐native species and human migration is inherently xenophobic, there are legitimate reasons to oppose the introduction of non‐native species (Simberloff, 2003; Simberloff *et al*., 2012, 2024; Richardson & Ricciardi, 2013) or unchecked migration (Sert & Erenler, 2021), while both the use of non‐native species (Sax *et al*., 2022) and human migration holds significant socio‐cultural and economic value (van Riemsdijk, Basford & Burnham, 2016; Adaçay, 2019).

There is often a reluctance to explore uncomfortable parallels between biological invasions and human migrations, likely due to a broader unease within academic circles for addressing topics that challenge established narratives. The pursuit of nuanced and complex understandings in the humanities and social sciences can sometimes overshadow straightforward, reality‐based comparisons. This reflects a broader academic trend where the detailed analysis of socio‐political dynamics may unintentionally distance critical perspectives from the practical realities often addressed in more practically grounded disciplines like engineering. Consequently, while the differences between non‐native species introductions and human migrations are indeed critically important, completely avoiding direct comparisons risks overlooking the broader systemic issues at play. For this reason, we need to explore the boundaries between invasion science and social sciences (currently with different backgrounds, languages, methods, and structures of thought) to inform professionals (individuals and groups) with a more integrated interdisciplinary perspective to address the problem. The academic inclination to distance these discussions from practical realities might inadvertently perpetuate the problems rather than solve them, suggesting that an integrated approach grounded in both scientific and practical understanding is essential.

There may be, however, untapped potential in interdisciplinary research to understand better both the effects of human migrations and of introduced non‐native species (Switzer & Angeli, 2016). Applying invasion science principles and models to human migration may offer intriguing insights while raising ethical and practical concerns. Moreover, while ecological models can inform migration policies by highlighting potential impacts, the unique complexities of human societies – such as legal rights, cultural integration, and economic motivations – necessitate distinct, tailored approaches. As such, examining non‐native species introductions through the framework used for studying human migration, often involving fields like sociology, anthropology, and political science, could provide new insights into managing these ecological events. Future discussions should explore how scientific frameworks can complement, rather than replace, human‐centric policies to address migration's multifaceted challenges.

## VI. CONCLUSIONS

(1) *Language and rhetoric*: the misuse of invasion science terminology in political and media discourse can foster xenophobia and discrimination, emphasising the importance of precise language in both scientific communication and public discussions. Particular care should be taken with terminology in invasion science to reduce misunderstandings and improve public and policy discourse.
(2) *Distinction between human migration and non‐native species introductions*: despite superficial similarities, human migration and non‐native species introductions involve fundamentally different processes driven by distinct socio‐economic, political, and biological factors, necessitating separate frameworks for analysis and management.
(3) *Historical context and cause*: the historical roots of migration and species introductions differ significantly, with human migration often driven by socio‐political factors like colonialism, economic disparities, and conflicts, while non‐native species introductions are closely tied to human activities like trade, globalisation, and environmental factors.
(4) *Ethical considerations*: applying invasion science principles to human migration can oversimplify complex socio‐political dynamics and dehumanise migrants, underscoring the need to consider the inherent human rights, sentience, morality, and agency of humans that do not apply to non‐native species.
(5) *Policy implications*: effective governance should distinguish terminology used for managing non‐native species and human migration, tailoring strategies to address the unique challenges of each phenomenon while avoiding harmful generalisations that can lead to misguided policies.
(6) *Interdisciplinary collaboration*: there is potential for interdisciplinary research between invasion science and social sciences to enrich our understanding of both non‐native species introductions and human migration, although ethical and practical distinctions must be carefully maintained.
